# A dual-reporter mouse for therapeutic discovery in Angelman syndrome

**DOI:** 10.1172/jci.insight.197028

**Published:** 2026-02-03

**Authors:** Hanna Vihma, Lucas M. James, Hannah C. Nourie, Audrey L. Smith, Siyuan Liang, Carlee A. Friar, Tasmai Vulli, Lei Xing, Dale O. Cowley, Alain C. Burette, Benjamin D. Philpot

**Affiliations:** 1 Department of Cell Biology and Physiology, Neuroscience Center,; 2 Animal Models Core,; 3 Department of Genetics, and; 4 Carolina Institute for Developmental Disabilities, University of North Carolina at Chapel Hill, Chapel Hill, North Carolina, USA.

**Keywords:** Genetics, Neuroscience, Drug screens, Imprinting, Neurodevelopment

## Abstract

Angelman syndrome is a neurodevelopmental disorder caused by loss of the maternal *UBE3A* allele, the sole source of UBE3A in mature neurons owing to epigenetic silencing of the paternal allele. Although emerging therapies are being developed to restore UBE3A expression by activating the dormant paternal *UBE3A* allele, existing mouse models for such preclinical studies have limited throughput and utility, creating bottlenecks for both in vitro therapeutic screening and in vivo characterization. To address this, we developed the *Ube3a*-INSG dual-reporter knockin mouse, in which an IRES-Nanoluciferase-T2A-Sun1-sfGFP (INSG) cassette was inserted downstream of the endogenous *Ube3a* stop codon. The INSG model preserves UBE3A protein levels and function while enabling 2 complementary allele-specific readouts: Sun1-sfGFP and Nanoluciferase. We show that Sun1-sfGFP, a nuclear envelope–localized reporter, enables single-cell fluorescence analysis, whole-brain light-sheet imaging, and nuclear quantification by flow cytometry. Further, Nanoluciferase supports high-throughput luminescence assays for sensitive pharmacological profiling in cultured neurons and noninvasive in vivo bioluminescence imaging for pharmacodynamic assessment. By combining scalable screening, cellular analysis, and real-time in vivo monitoring in a single model, the *Ube3a*-INSG dual-reporter mouse provides a powerful platform to accelerate therapeutic development centered on UBE3A.

## Abstract 2

Multimodal dual-reporter mouse accelerates Angelman syndrome therapeutic development through scalable cell-based screening, high-resolution whole-brain mapping, non-invasive live imaging, and sorting neurons with unsilenced paternal *Ube3a*.

## Introduction

Angelman syndrome (AS) is a rare neurodevelopmental disorder affecting approximately half a million individuals worldwide (1). Individuals with AS can have a normal lifespan, yet they face profound and lifelong challenges including severe motor dysfunction, intellectual disability, and an absence of speech, with epilepsy also commonly observed (2). These challenges create significant economic and social burdens for families and health care systems (3). AS is caused by disruption of the maternally inherited allele of the ubiquitin protein ligase E3A (*UBE3A*) gene (4–6). Both maternal and paternal copies of *UBE3A* are expressed in most cell types. However, in mature neurons, the paternal allele is epigenetically silenced by a long non-coding antisense transcript (*UBE3A-ATS*), leaving only the maternal allele to produce UBE3A (7).

Current standard-of-care practices for AS are limited to symptom management, with treatments such as antiepileptic drugs and behavioral therapies (8). Efforts to develop effective therapies have focused on restoring UBE3A protein levels in neurons. In particular, the unique parent-of-origin imprinting of *UBE3A* has prompted therapeutic strategies aimed at reactivating the dormant paternal allele. Several approaches have been developed to target *UBE3A-ATS* and restore UBE3A expression, including small molecules (9, 10), antisense oligonucleotides (ASOs) (11–15), artificial transcription factors (16), and CRISPR-based strategies such as gene editing, transcriptional interference by dCas9, and RNA targeting with Cas13 (17–20). Among these, ASO therapies have advanced the furthest, with multiple candidates currently undergoing clinical evaluation (ClinicalTrials.gov: NCT06617429, NCT06914609, NCT04428281).

The YFP-tagged UBE3A fusion knockin reporter mouse (RRID:IMSR_JAX:017765) has enabled allele-specific visualization of UBE3A expression and has served as a valuable screening platform for unsilencing compounds (9, 10, 21, 22). However, this model has notable limitations: The YFP signal typically requires immunostaining for reliable detection, making quantitative analyses labor-intensive and lower throughput. In addition, UBE3A-YFP exhibits diffuse nuclear and cytoplasmic localization, which complicates single-cell quantification in densely packed tissues.

To address these limitations, we developed the *Ube3a*-INSG dual-reporter knockin mouse model for sensitive, quantitative monitoring of UBE3A expression from either parental allele at single-cell resolution. The INSG allele encodes 2 reporters. Nanoluciferase (Nluc) enables high-throughput luminescence-based quantification and noninvasive in vivo bioluminescence imaging. Sun1-sfGFP, a nuclear envelope–anchored superfolder GFP (sfGFP) reporter, supports cellular-level quantitative immunofluorescence and flow cytometry. This dual-reporter design allows applications spanning high-throughput in vitro screening to high-resolution, whole-brain evaluation of biodistribution and pharmacodynamic effects in vivo. Here, we demonstrate the utility of this system in accelerating therapeutic development efforts centered on UBE3A.

## Results

### Generation of the Ube3a-INSG reporter mice.

**Figure 1:**
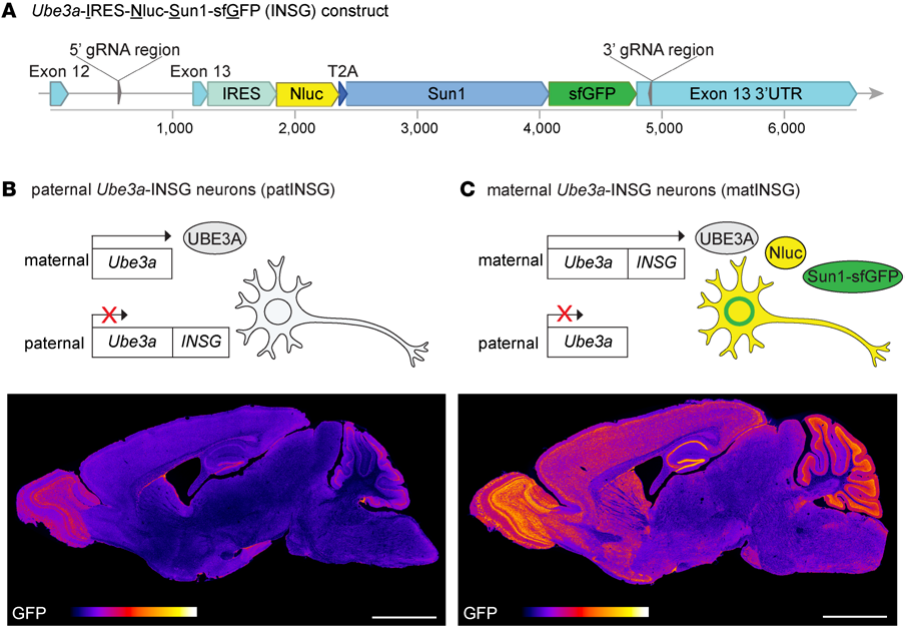
Generation and allele-specific expression of the *Ube3a-IRES-Nluc-Sun1-sfGFP* (*INSG*) reporter allele. (**A**) Schematic of the INSG construct and guide RNAs (gRNAs) used for pronuclear microinjection. The 3,566 bp INSG reporter cassette was inserted downstream of the *Ube3a* stop codon in exon 13. It includes an internal ribosomal entry site (IRES), nanoluciferase coding sequence (Nluc), 2A self-cleaving peptide from *Thosea asigna* virus capsid protein (T2A), and Sun1 nuclear envelope localization domain (Sun1) fused to the N-terminus of superfolder GFP (sfGFP). The 5′ and 3′ gRNA target site regions used for the homologous recombination are shown in *Ube3a* intron 12 and 3′-UTR, respectively. (**B**) Schematic of the paternal allele expression of the *Ube3a*-INSG transgene (patINSG), where *Ube3a*-INSG is paternally inherited and silenced in neurons, resulting in maternal-only UBE3A expression in most mature neurons. The sagittal section from a postnatal day 30 (P30) patINSG brain shows low overall GFP signal, with some residual expression in the olfactory bulb, newborn neurons in the dentate gyrus, and cerebellar cortex. (**C**) Schematic of the maternal allele expression of the *Ube3a*-INSG transgene (matINSG), where *Ube3a*-INSG is maternally inherited, enabling expression of UBE3A, Nluc, and Sun1-sfGFP from the maternal allele. The corresponding sagittal section shows strong GFP labeling in the cerebrum and cerebellum, with lower signal in the brainstem. Scale bars: 2 mm. Fire gradient used for visualization.

To generate the *Ube3a*-INSG reporter mouse line, we used CRISPR/Cas9–mediated genome editing to insert a 3,566 bp reporter cassette immediately downstream of the *Ube3a* stop codon located in exon 13 (Figure 1A). The INSG reporter cassette includes an internal ribosomal entry site (IRES), an Nluc coding sequence, a T2A self-cleaving peptide from the *Thosea asigna* virus, and a Sun1-sfGFP fusion gene. The sfGFP is fused to an N-terminally truncated mouse (*Mus musculus*) Sun1 sequence, which directs the fusion protein to the nuclear envelope (23). The 2 reporters encoded by the INSG allele provide complementary strategies for monitoring *Ube3a* expression.

Due to epigenetic silencing of the paternal *Ube3a* allele, mice with the INSG transgene inherited paternally (patINSG) show minimal reporter expression in the brain at postnatal day 30 (P30), an age by which paternal *Ube3a* is silenced in neurons (Figure 1B) (22). Further, regions undergoing active neurogenesis — the subgranular zone of the dentate gyrus and the subventricular zone — along with areas where postnatal neurogenesis has recently ceased, such as the olfactory bulb and cerebellum, display sfGFP expression, consistent with their maturation status. In contrast, maternal inheritance of the INSG transgene (matINSG) leads to strong coexpression of endogenous UBE3A along with the Nluc and Sun1-sfGFP reporters (Figure 1C). Sagittal brain sections from matINSG mice show widespread GFP labeling that closely matches UBE3A expression, with the highest signals in the cerebrum (including cortex, hippocampus, basal ganglia, and olfactory bulb) and cerebellum, and the lowest in the brainstem (Figure 1B).

### INSG insertion preserves UBE3A protein levels and function.

**Figure 2:**
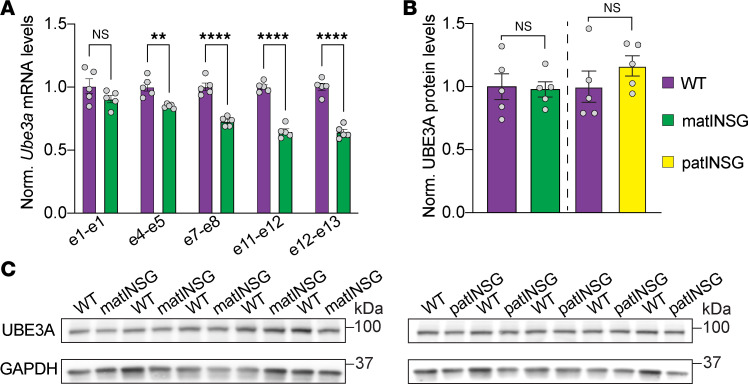
Insertion of INSG reporter cassette into the *Ube3a* 3′-UTR preserves UBE3A protein levels. Brains from P90 mice were split so that one hemisphere was used for RNA extraction and the opposite hemisphere for protein analysis. Data from both assays were normalized to WT littermate controls. (**A**) Quantification of *Ube3a* mRNA normalized to *Eif4a2* in WT and matINSG using primer sets targeting the indicated exons (2-way ANOVA with Bonferroni’s post hoc test). (**B**) Quantification of UBE3A protein levels in WT, matINSG, and patINSG mice normalized to GAPDH (2-tailed *t* test). (**C**) Representative Western blots comparing WT versus matINSG and WT versus patINSG. Each data point represents an individual animal (*N* = 5 per genotype; all males), with data shown as means ± SEM. **P < 0.005, ****P < 0.0001. Detailed statistical comparisons are provided in the Supporting Data Values. Norm., normalized; e, exon.

Previous studies have shown that adding YFP or other protein linkers to the C-terminus of UBE3A can interfere with its expression or enzymatic activity, as reported for the *Ube3a*-YFP mouse model (24–26). In contrast, the INSG design avoids direct modification of the UBE3A protein. Instead, Nluc and Sun1-sfGFP are translated as independent polypeptides from the same transcript, rather than as fusion proteins. To assess whether inserting the INSG cassette downstream of *Ube3a* affects endogenous expression, we quantified *Ube3a* mRNA levels using primer sets located in exon 1 and within exons 4–5, 7–8, 11–12, and 12–13 (Figure 2A). These assays showed a progressive decrease in transcript abundance toward the 3′ end of the gene. Similar, but less pronounced, reductions were observed in patINSG and patYFP mice compared with wild-type (WT) mice (Supplemental Figure 1, A and B; supplemental material available online with this article; https://doi.org/10.1172/jci.insight.197028DS1), suggesting that insertion into the 3′-UTR may influence transcript stability or processing. Despite these mRNA changes, Western blot analysis showed that UBE3A protein levels were unchanged across genotypes (Figure 2, B and C), indicating that the insertion of the INSG cassette does not measurably impair UBE3A protein expression in either matINSG or patINSG mice.

**Figure 3:**
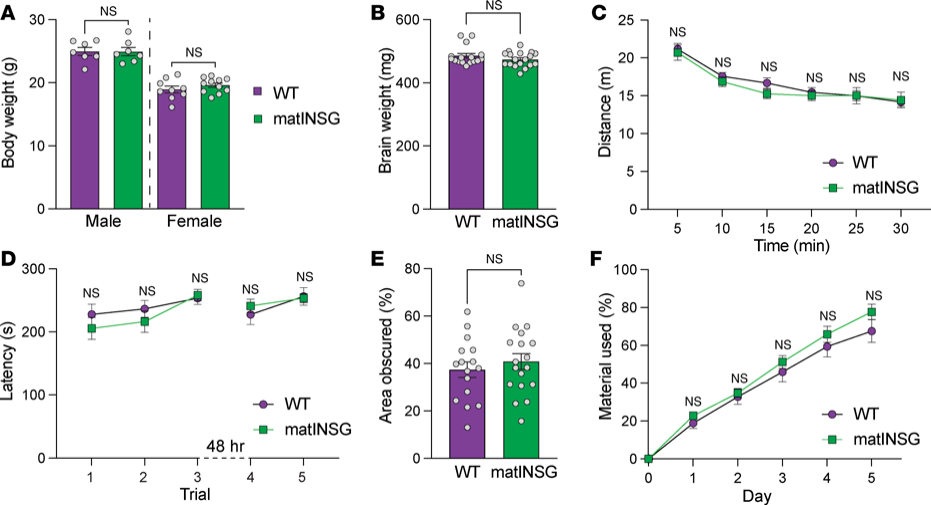
Insertion of INSG reporter cassette into the *Ube3a* 3′-UTR preserves UBE3A protein function. (**A** and **B**) Body weight (**A**) and brain weight (**B**) did not differ significantly between WT and matINSG mice (2-tailed *t* test). (**C**–**F**) Behavioral phenotyping shows no significant differences between WT and matINSG mice in distance traveled in the open field (2-way repeated-measures ANOVA, Bonferroni’s post hoc test) (**C**), latency to fall on an accelerating rotarod (2-way repeated-measures ANOVA, Bonferroni’s post hoc test) (**D**), percentage of marble area obscured by bedding in a 30-minute test session (2-tailed *t* test) (**E**), and nesting material used over a 5-day nest-building assay (2-way repeated-measures ANOVA, Bonferroni’s post hoc test) (**F**). Body weight and brain weight were measured at P90. Behavioral tests were conducted on the same cohort beginning around P70 (WT, *N* = 18 [7 males, 11 females]; matINSG, *N* = 16 [7 males, 9 females]) and performed in the order shown. Each data point represents an individual animal, with data shown as means ± SEM. Detailed statistical comparisons are provided in the Supporting Data Values.

To confirm whether inserting the INSG reporter cassette into the 3′-UTR of *Ube3a* affects UBE3A protein function, we performed anatomical and behavioral analyses in *Ube3a*-INSG mice (Figure 3). Since excessive adult weight gain and microcephaly are well-documented phenotypes in *Ube3a* maternal deletion AS mouse models (27–29), we evaluated body and brain weights at P90. MatINSG mice had body and brain weights similar to those of their WT littermates (Figure 3, A and B, respectively), indicating that the insertion of the INSG cassette does not disrupt overall growth or neurodevelopment. We then assessed whether the INSG insertion changes neurological function by testing matINSG mice for behavioral deficits commonly observed in AS models (30). Consistent with preserved UBE3A protein levels and function, matINSG mice showed no significant differences from WT littermates in locomotor activity (open field distance, Figure 3C), motor coordination (accelerating rotarod, Figure 3D), or species-typical behaviors (marble burying, Figure 3E, and nest building, Figure 3F). 

The maintained UBE3A protein levels and lack of anatomical and behavioral deficits in comparison with WT mice strongly support that inserting the INSG cassette does not compromise the functional integrity of UBE3A expressed from the INSG allele.

### Sun1-sfGFP enables quantitative cellular analysis of UBE3A imprinting and paternal allele reactivation.

**Figure 4:**
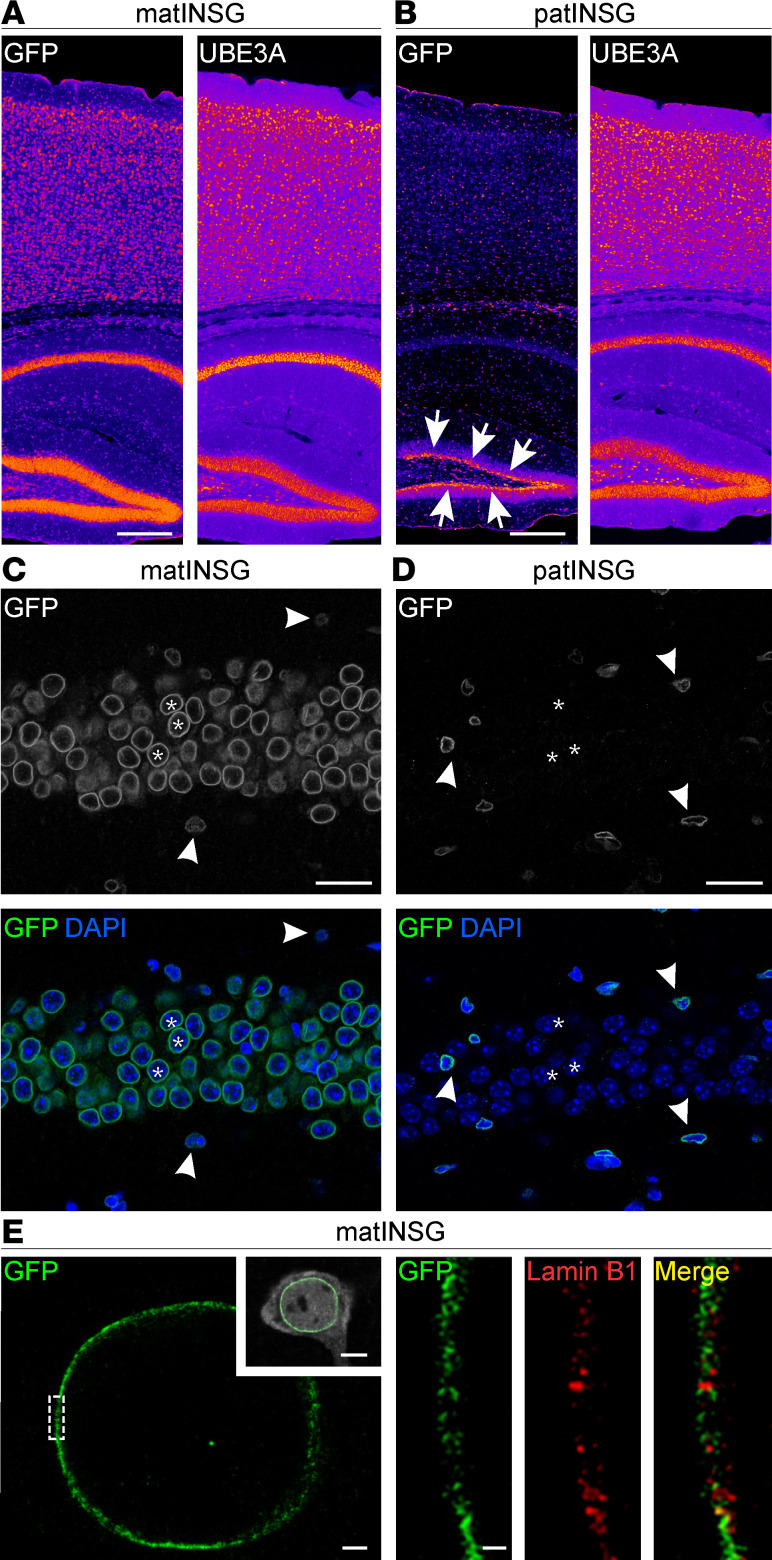
Validation of Sun1-sfGFP as an allele-specific, nuclear envelope–localized reporter of UBE3A expression. (**A** and **B**) Hippocampal and cortical regions in P30 matINSG and patINSG mice. (**A**) In matINSG brain, GFP (left) and UBE3A (right) levels are closely matched. (**B**) In patINSG brain, GFP (left) is sparser than UBE3A (right), with a strong signal only in immature neurons of the dentate gyrus subgranular zone (arrows). Fire gradient used for visualization. (**C** and **D**) High magnification of the hippocampal CA1 region. (**C**) In matINSG, DAPI (blue, bottom) and GFP staining (gray, top; green, bottom) show expression in pyramidal neurons (asterisks) and smaller cells outside the layer (arrowheads). (**D**) In patINSG, only scattered small cells, presumably glia, are GFP positive (gray, top; green, bottom) (arrowheads), while pyramidal neurons lack staining (asterisks). In both genotypes, GFP localizes to the nuclear membrane, consistent with Sun1 fusion. (**E**) STED microscopy of a matINSG neuron showing Sun1-sfGFP localization at the nuclear envelope. GFP from the boxed region (left) is shown at higher magnification to the right, alongside lamin B1 and the merged image, confirming membrane localization. The inset in **E** shows an overview of the same cell stained for NeuN (gray) and GFP (green). Scale bars: 250 μm (**A** and **B**); 25 μm (**C** and **D**); 1 μm (**E**); 5 μm (inset in **E**); 200 nm (right panel in **E**).

Next, we examined how reliably Sun1-sfGFP reports UBE3A expression. We compared GFP and UBE3A immunolabeling patterns in matINSG and patINSG brains at P30. Our focus was on the hippocampus, where strong GFP labeling in the pyramidal cell layer of matINSG mice was substantially diminished in patINSG mice (Figure 4, A and B, respectively). The GFP signal remained high in the subgranular zone of the dentate gyrus, at the border between the granule cell layer and the hilus (arrows in Figure 4B), consistent with known paternal *Ube3a* expression in newly formed neurons (22).

Higher resolution imaging of the CA1 hippocampal field revealed distinct Sun1-GFP labeling patterns dependent on reporter parent of origin (Figure 4, C and D). In matINSG brains, DAPI counterstaining showed intense GFP signal localized to the nuclear envelope of both pyramidal neurons and scattered smaller cells (Figure 4C). In contrast, the patINSG brain exhibited markedly reduced GFP staining confined to the nuclear membrane of a few smaller cells, presumed to be glial cells, with pyramidal neurons largely devoid of signal (Figure 4D). We used stimulated emission depletion (STED) microscopy to further validate the nuclear membrane localization of the GFP signal. Double labeling for GFP and lamin B1, a canonical nuclear envelope marker (31), revealed that GFP staining was interspersed with lamin B1 (Figure 4E). This pattern confirmed the expected nuclear envelope localization of the Sun1-sfGFP fusion protein (32).

**Figure 5:**
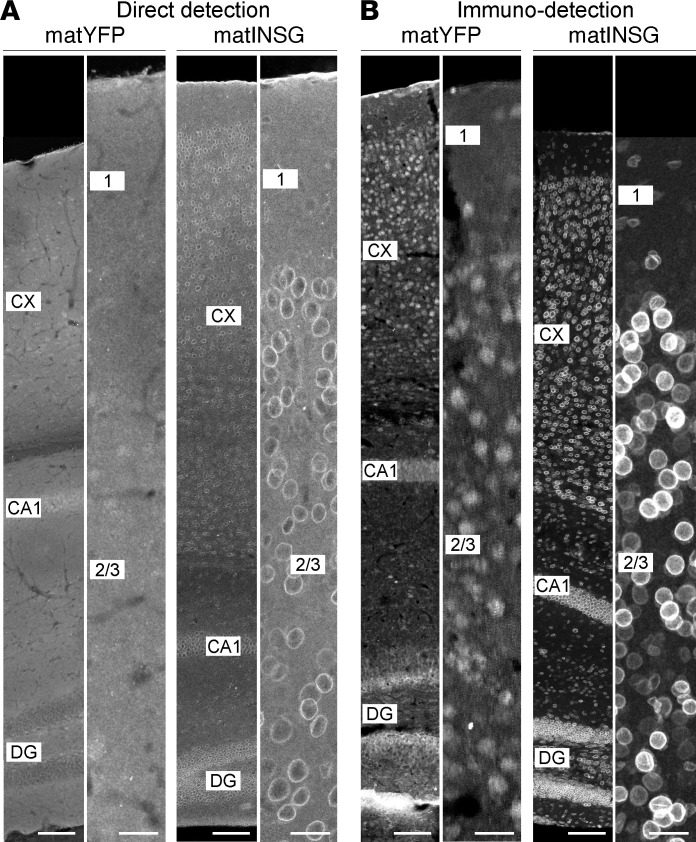
The matINSG Sun1-sfGFP reporter provides superior signal and cellular resolution compared with matYFP. (**A**) Direct fluorescence detection in matYFP and matINSG mice at P13 showing the neocortex (CX) and hippocampal region (CA1 region and dentate gyrus [DG]). No specific signal is readily visible in matYFP mice beyond background fluorescence, whereas matINSG mice exhibit a weak but clearly detectable nuclear-localized sfGFP signal. (**B**) Immunodetection using GFP antibody in matYFP and matINSG mice. The INSG reporter demonstrates superior cellular resolution with distinct nuclear labeling, while YFP shows more diffuse cytoplasmic staining. Higher-magnification images of neocortex layers 1 to 3 (right panels in **A** and **B**) highlight individual cells and the improved signal-to-background ratio in matINSG mice. Scale bars: 25 μm (left micrographs); 100 μm (right micrographs).

We then directly compared the INSG model with the *Ube3a*-YFP reporter line by performing a side-by-side comparison of matINSG and matYFP brains at P13, using both native fluorescence and immunodetection (Figure 5). The Sun1-sfGFP reporter produced markedly superior signal resolution, even without signal enhancement (Figure 5A), yielding a sharp, ring-like pattern at the nuclear envelope that offered clear definition (Figure 5). In contrast, native YFP fluorescence was barely detectable in brain tissue (Figure 5A). Following antibody enhancement, the cytoplasmic YFP signal in *Ube3a*-YFP mice improved but appeared diffuse and poorly resolved in dense brain tissue, making cellular quantification more difficult (Figure 5B). These findings highlight the advantages of the INSG model for high-resolution imaging and accurate, cell-specific quantification of allele-specific UBE3A expression.

**Figure 6:**
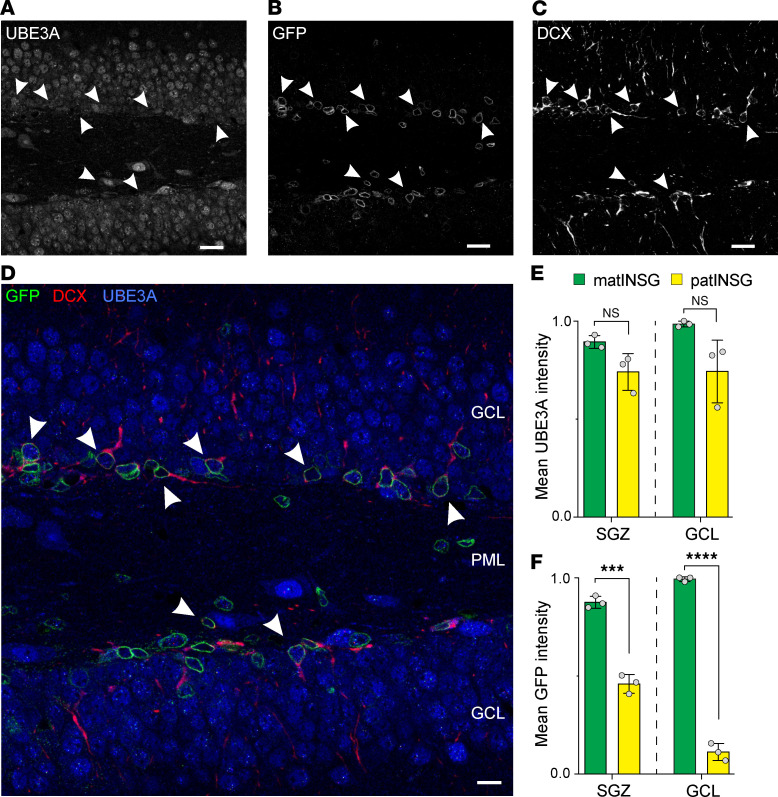
Sun1-sfGFP is expressed in immature neurons in the patINSG hippocampus. (**A**–**C**) Triple immunofluorescence staining of the dentate gyrus in P30 patINSG mice for UBE3A (**A**), Sun1-sfGFP (GFP) (**B**), and the immature neuron marker doublecortin (DCX) (**C**). (**D**) The merged image highlights that, in contrast to the widespread detection of UBE3A, GFP expression is restricted chiefly to DCX-positive immature neurons (arrowheads) located in the subgranular zone. (**E** and **F**) Mean intensity of UBE3A (**E**) and GFP (**F**) in the subgranular zone (SGZ; defined by DCX staining) and in the granule cell layer (GCL) of the dentate gyrus in matINSG and patINSG mice (*N* = 3 animals per group). Data are presented as mean ± SEM. One-tailed unpaired *t* test (***P < 0.001, ****P < 0.0001). Scale bars: 20 μm (**A**–**C**); 10 μm (**D**). PML, polymorphic layer.

To highlight this utility, we performed quantitative analyses of UBE3A in the densely packed hippocampus. To identify the strongly labeled cells within the subgranular zone (SGZ) of the dentate gyrus, we carried out triple immunolabeling for GFP, UBE3A, and the microtubule-associated protein doublecortin (DCX) (Figure 6). In the adult dentate gyrus, DCX is expressed almost exclusively in developing neurons, serving as a reliable marker of neurogenesis (33). We observed that most SGZ cells with high GFP intensity coexpressed both DCX and UBE3A (Figure 6), consistent with these being immature neurons that biallelically express *Ube3a*. We then compared the mean GFP and UBE3A fluorescence intensities between DCX-positive SGZ cells and neurons in the surrounding granule cell layer (GCL) (Figure 6, E and F). In patINSG mice, GFP intensity was significantly higher in the SGZ than in the GCL, whereas in matINSG mice, strong GFP signals were present throughout the GCL. These findings support that paternal *Ube3a* expression is developmentally regulated and persists longer in immature neurons, as shown by sustained reporter expression in DCX-positive cells. Although earlier studies have reported paternal *Ube3a* expression in immature neurons (22, 34), to our knowledge, this is the first quantitative in vivo analysis of this developmental pattern.

**Figure 7:**
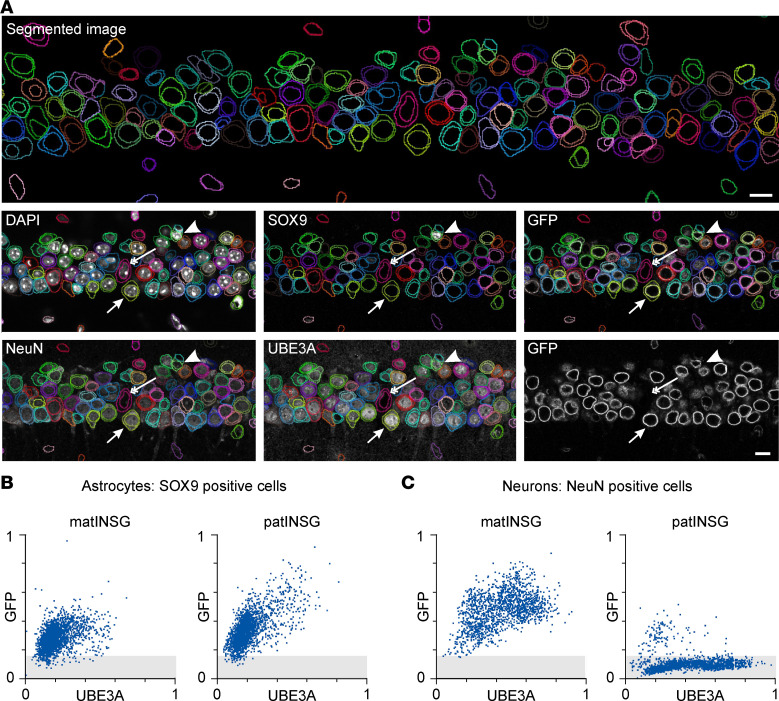
Relationship between UBE3A and Sun1-sfGFP labeling in individual cells in matINSG versus patINSG mice. (**A**) An illustration of the effectiveness of the deep learning algorithm used to quantify UBE3A and Sun1-sfGFP expression at single-cell resolution, even in densely packed areas like the hippocampal CA1 region. For easier visualization, each cell is outlined with a randomly assigned color to indicate both nuclear and cytoplasmic boundaries. Each of the 6 smaller panels overlays the cell segmentation results with 1 of the 5 input channels (DAPI, SOX9, GFP, NeuN, or UBE3A) used for segmentation, while the bottom right panel shows only the GFP channel without cell segmentations. The arrows point to a likely pyramidal neuron that expresses GFP, UBE3A, and the neuronal marker NeuN but not the astrocyte marker SOX9. The arrowheads indicate a small cell that is GFP, UBE3A, and SOX9 positive, but NeuN negative. The double arrows identify an outlier cell lacking all tested markers. (**B** and **C**) Scatterplots depicting the relationship between GFP and UBE3A fluorescence levels in individual cells within the hippocampal CA1 region at P30. (**B**) In SOX9-positive astrocytes, GFP and UBE3A intensities strongly correlate in matINSG and patINSG mice. (**C**) In NeuN-positive neurons, there is a strong correlation between GFP and UBE3A intensities in matINSG mice but not patINSG mice, apart from a small subset of cells, as most neurons in patINSG mice display GFP levels at background levels. The gray-shaded area marks the range of background GFP fluorescence observed in UBE3A-positive neurons from patINSG samples (**C**, right), which lack Sun1-sfGFP expression. This reference range is shown across all plots to facilitate comparison across genotypes and cell types. Scale bars: 10 μm.

To validate that the cellular GFP reporter accurately reflects endogenous UBE3A, we examined expression in specific cell types following quintuple immunolabeling for GFP, UBE3A, and markers for astrocytes (SOX9), neurons (NeuN), and nuclei (DAPI) in the hippocampal CA1 field (Figure 7). In matINSG mice, robust GFP and UBE3A labeling was observed in both astrocytes (NeuN^–^/SOX9^+^) and mature neurons (NeuN^+^/SOX9^–^) (Figure 7, B and C, respectively). Analysis of 1,572 neurons showed high GFP expression (x = 0.452 ± 0.108; mean ± SD) with a strong linear correlation to UBE3A levels (Pearson’s *r* = 0.559, Spearman’s ρ = 0.549, *P* < 0.0001). Astrocytes, which express UBE3A from both alleles, displayed more moderate GFP expression (*n* = 2,306, x = 0.253 ± 0.075; mean ± SD) with a significant correlation to UBE3A (*r* = 0.382, ρ = 0.442, *P* < 0.0001). In patINSG mice, neurons exhibited markedly reduced GFP labeling (*n* = 2,059, x = 0.078 ± 0.053; mean ± SD), with only a few exceptions, while UBE3A labeling remained consistent (x = 0.354 ± 0.183; mean ± SD) (Figure 7C). Conversely, astrocytes continued to display strong labeling for both UBE3A and GFP, with the strongest correlation between the two (*n* = 2,222, *r* = 0.602, ρ = 0.607, *P* < 0.0001) (Figure 7B). These findings confirm biallelic *Ube3a* expression in glial cells, consistent with prior studies (21, 22, 35, 36), and extend these observations by providing a quantitative, cell type–specific in vivo readout using a genetically encoded reporter.

**Figure 8:**
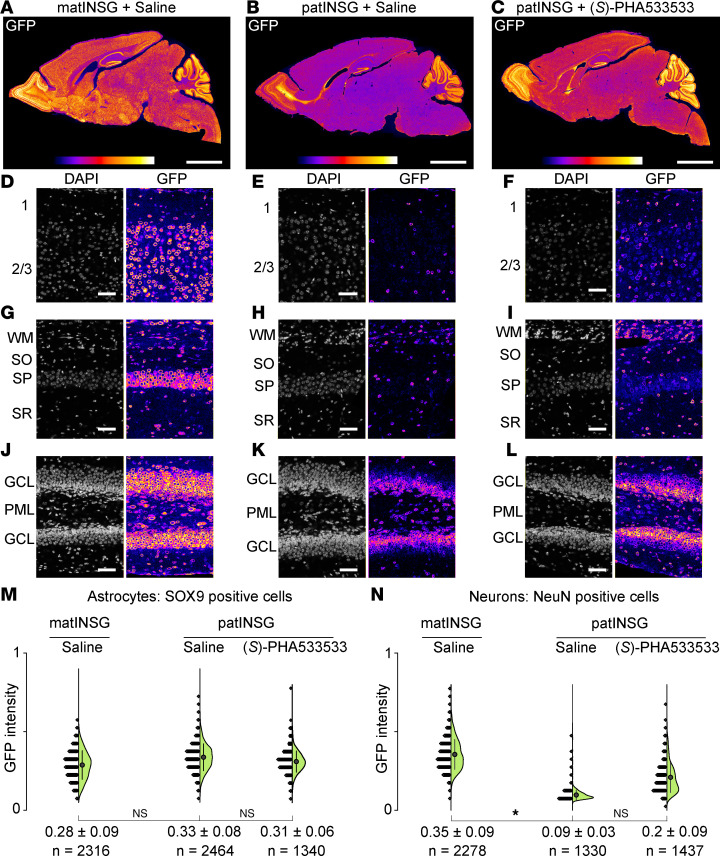
Paternal *Ube3a* unsilencing effect of (*S*)-PHA533533 in mouse brain measured using the Sun1-sfGFP reporter system. MatINSG and patINSG mice were treated intraperitoneally at P11 with either saline or (*S*)-PHA533533 (2 mg/kg), and brains were analyzed at P13. UBE3A expression was assessed via GFP immunostaining. (**A**–**C**) Sagittal brain sections visualized using a fire lookup table: saline-treated matINSG (**A**), saline-treated patINSG (**B**), and patINSG treated with (*S*)-PHA533533 (**C**). (**D**–**L**) Double staining for GFP (fire lookup table) and DAPI (white). (**D**–**F**) Layers 1 and 2/3 of the neocortex. (**G**–**I**) Hippocampal CA1 region highlighting white matter (WM), stratum oriens (SO), stratum pyramidale (SP), and stratum radiatum (SR). (**J**–**L**) Dentate gyrus, showing granule cell layer (GCL) and polymorphic layer (PML). (**M** and **N**) Violin plots of GFP intensity in CA1 hippocampal cells (*N* = 3 mice per condition; individual cells shown as *n*): (**M**) SOX9-positive astrocytes show similar GFP levels across groups. (**N**) NeuN-positive neurons show reduced GFP in saline-treated patINSG versus matINSG, with partial rescue after (*S*)-PHA533533 treatment. Data are mean ± SD. Statistical analysis by 1-way ANOVA with Bonferroni’s post hoc test. *P < 0.05. Scale bars: 2 mm (**A**–**C**); 50 μm (**D**–**L**).

Next, we tested the ability of Sun1-sfGFP to report in vivo reactivation of paternal *Ube3a* using (*S*)-PHA533533, a small molecule previously shown to unsilence paternal *Ube3a* after peripheral administration via a currently unidentified mechanism (10, 37). P11 patINSG mice received a single 2 mg/kg dose of (*S*)-PHA533533, while saline-treated matINSG and patINSG mice served as positive and negative controls, respectively (Figure 8). Analysis of Sun1-GFP expression at P13, 48 hours after treatment, in sagittal brain sections showed baseline GFP expression in patINSG brains compared with widespread signals in matINSG controls (Figure 8, A and B). In contrast, patINSG mice treated with (*S*)-PHA533533 showed visible partial reactivation of the GFP reporter across the brain (Figure 8C). Confocal imaging suggested that this reactivation happened within neuronal populations. This was especially in regions where neurons are readily distinguished by their size, shape, and location, including in superficial neocortical layers 1–2/3 (Figure 8, D–F), the pyramidal cell layer in the hippocampal CA1 field (Figure 8, G–I), and the dentate gyrus granule cell layer (Figure 8, J–L). This specific partial reactivation of paternal *Ube3a* by (*S*)-PHA533533 was further confirmed by costaining with NeuN and SOX9 (Figure 8, M and N). Assessment of GFP intensity in nuclei across all 3 layers of the hippocampal CA1 region showed that SOX9-positive astrocytes maintained consistent GFP levels (Figure 8M) with no significant differences between matINSG and patINSG mice, regardless of treatment. These findings are consistent with biallelic *Ube3a* expression in glial cells, as previously reported in mice and nonhuman primates (21, 22, 35, 36), as well as (*S*)-PHA533533 not affecting *Ube3a* expression in biallelically expressing cell types (10). In contrast, we observed a significant difference in GFP intensity between saline-treated matINSG and patINSG mice in NeuN-positive neurons, reflecting paternal allele silencing (Figure 8N). Treatment of patINSG mice with 2 mg/kg (*S*)-PHA533533 increased GFP levels in NeuN-positive neurons, indicating partial reactivation of the paternal *Ube3a* allele (Figure 8N).

These data demonstrate that Sun1-sfGFP functions as a highly reliable, allele-specific reporter of UBE3A expression at regional and cellular levels. It accurately reflects *Ube3a* imprinting biology in the *Ube3a*-INSG mouse in vivo and sensitively reports pharmacologically induced expression changes, demonstrating its ability to assess the pharmacodynamics of unsilencing approaches. The strict confinement of GFP signal to the nuclear envelope facilitates quantitative single-cell analysis in complex tissues.

### INSG reporter enables whole-brain imaging of UBE3A expression.

**Figure 9:**
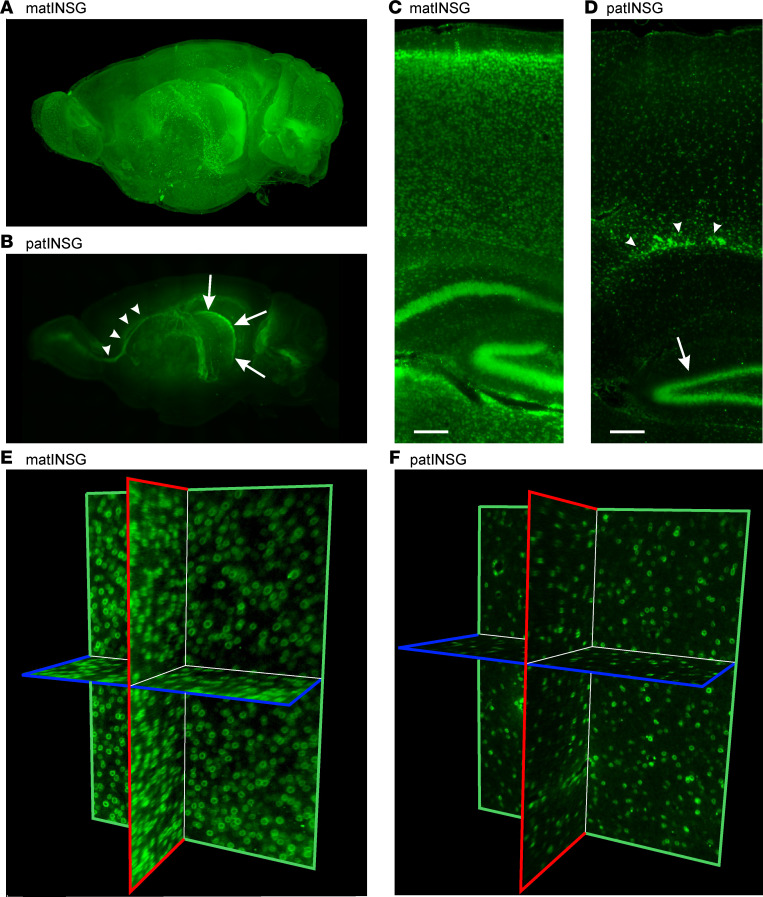
Brain-wide imaging of the Sun1-sfGFP reporter using LSFM. (**A** and **B**) Maximum-intensity projections of left hemispheres from P30 matINSG (**A**) and patINSG (**B**) brains labeled with GFP using iDISCO and visualized with 3D LSFM. The matINSG brain shows robust, widespread GFP expression, whereas patINSG shows lower overall expression, with the strongest labeling in the rostral migratory stream (arrowheads) and dentate gyrus (arrows). (**C** and **D**) Virtual 25 μm slices from **A** and **B**. (**C**) The matINSG brain shows a strong GFP signal across cortical layers and hippocampus, particularly in the pyramidal cell layer and dentate gyrus. (**D**) In contrast, the patINSG shows markedly reduced labeling, with prominent expression in the subventricular zone (arrowheads) and subgranular zone of the dentate gyrus (arrow). (**E** and **F**) Single light-sheet images in *XY*, *XZ*, and *YZ* planes from the neocortex of **A** and **B**, showing single-cell resolution. In both matINSG and patINSG brains, individual GFP-positive cells are identifiable, with resolution sufficient to show the nuclear localization of the Sun1-sfGFP reporter. The matINSG neocortex exhibits markedly higher GFP-positive cell density compared with patINSG, with the patINSG neocortex predominantly showing labeling of small nuclei, presumably of glia. Scale bars: 150 μm (**C** and **D**).

Assessing brain-wide UBE3A distribution is crucial for evaluating the efficacy of therapeutics aimed at reactivating paternal *Ube3a* expression. However, direct immunodetection of endogenous UBE3A in tissue-cleared whole brains proved challenging in our hands. Thus, we explored whether Sun1-GFP could overcome this problem by testing whether the Sun1-sfGFP reporter could enable visualization of UBE3A-expressing nuclei in the intact brain using light-sheet fluorescence microscopy (LSFM) (Figure 9). We used the iDISCO clearing protocol with GFP immunolabeling in P30 INSG brains (38). The Sun1-sfGFP reporter provided robust and comprehensive labeling throughout the entire brain, overcoming the limitations of direct UBE3A detection (Figure 9). MatINSG brains exhibited widespread GFP signal faithfully reproducing known UBE3A expression patterns (Figure 9, A–C), while patINSG brains exhibited markedly reduced GFP signal. However, residual GFP labeling in patINSG brains was confined to regions of known persistent UBE3A expression, such as neurogenic zones including the rostral migratory stream, which is difficult to assess thoroughly in individual sections, and the dentate gyrus (Figure 9, B and D, respectively). High-resolution imaging in neocortical regions confirmed that Sun1-sfGFP enables visualization of individual nuclei throughout the brain volume, with its characteristic nuclear envelope localization providing single-cell resolution (Figure 9, E and F).

### Dual Nluc and Sun1-GFP reporters enable high-throughput screening and flow cytometry analysis of allele-specific Ube3a expression.

**Figure 10:**
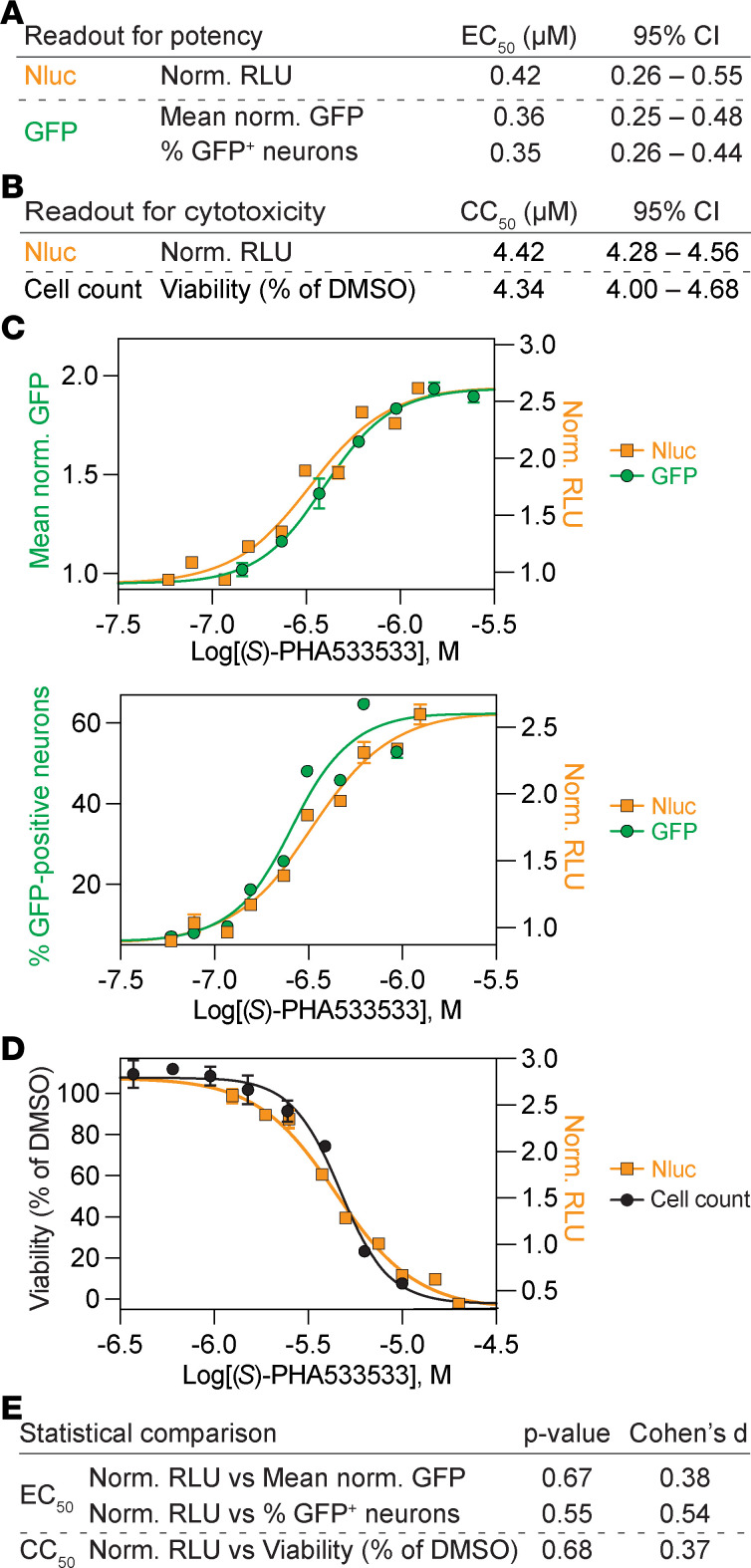
GFP and Nluc reporters provide concordant pharmacological readouts for measuring changes in allele-specific *Ube3a* expression in mouse primary neurons from patINSG and matINSG mice. (**A**–**D**) Pharmacological profiles for paternal *Ube3*a unsilencing in patINSG neurons treated with (*S*)-PHA533533 were assessed using luciferase- and immunofluorescence-based readouts. (**A**) EC_50_ values from 3 independent readouts — normalized relative light units (RLU), mean GFP fluorescence, and percentage GFP-positive neurons — show nearly identical potencies with overlapping 95% confidence intervals (CIs). (**B**) CC_50_ values from RLU and viability (percentage surviving neurons) also show overlapping 95% CIs. (**C** and **D**) Representative dose-response curves illustrate close agreement between Nluc and GFP readouts (**C**) and between Nluc and cell count–based cytotoxicity (**D**). (**E**) EC_50_ and CC_50_ values between GFP and Nluc assays showed no significant differences. All assays were performed in quadruplicate in 3 independent experiments (*N* = 3). Statistical analysis by 2-tailed *t* tests (**E**).

Previous screening efforts to identify compounds that unsilence paternal *Ube3a* relied on labor-intensive and costly high-content imaging workflows spanning multiple days (9, 10). The *Ube3a*-INSG reporter mouse was designed to enable robust, high-throughput in vitro screening via quantitative luminescence assays, made possible by the integrated Nluc reporter. To test this capability, we compared pharmacological profiles of the *Ube3a* unsilencer (*S*)-PHA533533 in primary neurons derived from patINSG mice using 2 complementary readouts: Nluc luminescence and immunocytochemistry-based (ICC-based) Sun1-sfGFP analysis (Figure 10, A–D). Efficacy measurements (half-maximal effective concentration [EC_50_]) derived from Nluc luminescence, mean GFP fluorescence, and the percentage of GFP-positive neurons were nearly identical, with overlapping 95% confidence intervals (Figure 10, A and C). Similarly, toxicity assessments (half-maximal cytotoxic concentration [CC_50_]) based on Nluc readout or by direct neuronal cell counting closely aligned (Figure 10, B and D). As expected, dose-response curves were highly similar across methods (Figure 10, C and D). Statistical comparisons further suggested no differences in potency and toxicity estimates between methods (Figure 10E), with small to medium Cohen’s *d* effect sizes, a standardized measure of difference between 2 means that accounts for variability in the data (39). Importantly, the EC_50_ values derived from the INSG model closely matched those previously obtained using ICC in *Ube3a*-YFP neurons (10), despite the difference in detection method and reporter. Additionally, we sought to validate our Nluc system using another recently reported unsilencer, octanoic acid, a medium-chain fatty acid recently reported to improve behavioral phenotypes in AS mice (40). However, we observed no increase in Nluc signal in patINSG neurons following treatment with octanoic acid (Supplemental Figure 2A), whereas our positive control, (*S*)-PHA533533, robustly unsilenced paternal *Ube3a* in the same assay (Supplemental Figure 2B). To confirm that this lack of unsilencing was not due to difference in reporter model, we dosed patYFP neurons with octanoic acid, but were again unable to detect the previously reported unsilencing of paternal *Ube3a* (Supplemental Figure 2C). Together, these results demonstrate that the Nluc-based assay provides pharmacological data comparable to ICC for screening paternal *Ube3a*-unsilencing small molecules, while offering substantially higher throughput.

**Figure 11:**
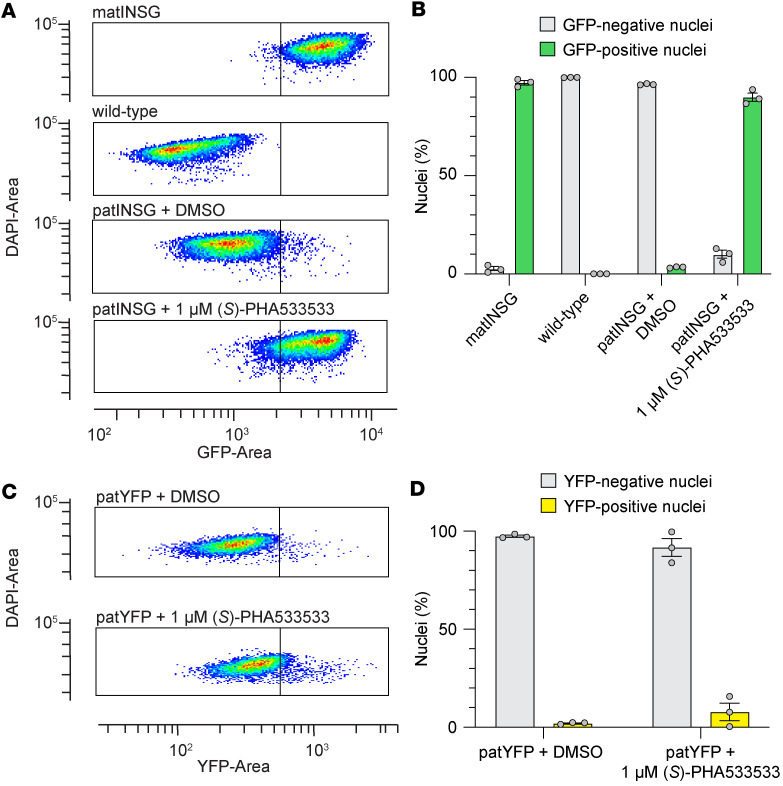
Sun1-GFP reporter enables flow cytometry for detecting paternal *Ube3a* unsilencing. (**A** and **B**) Flow cytometry was performed to assess the relative fluorescence of GFP/YFP in nuclei isolated from cultured neurons. (**A**) Representative density pseudocolored dot blots from sorted nuclei pools of cultured neurons derived from matINSG, WT, and patINSG mice, with patINSG neurons treated with either DMSO or 1 μM (*S*)-PHA533533 for 72 hours. The boxes indicate the gating boundaries that delineate the GFP-positive and GFP-negative populations. (**B**) Quantification of the percentage of GFP-positive and GFP-negative nuclei across samples. (**C**) Representative density pseudocolored dot blots from sorted nuclei pools of cultured neurons derived from patYFP mice treated with DMSO or 1 μM (*S*)-PHA533533 for 72 hours. (**D**) Quantification of the percentage of YFP-positive and YFP-negative nuclei across samples. Data are represented as mean ± SEM, where each data point corresponds to an independent well (10,000 nuclei analyzed per well).

To further evaluate the utility of the INSG system, we tested whether the Sun1-GFP reporter, which is anchored to the outer nuclear membrane, could be used for flow cytometry–based analysis. Nuclei isolated from WT, matINSG, and patINSG neurons treated with 1 μM (*S*)-PHA533533 or DMSO for 72 hours were stained with DAPI and analyzed for GFP fluorescence (Figure 11, A and B). As expected, nuclei from matINSG cultures showed strong GFP fluorescence compared with WT controls. Fluorescence distributions from WT and matINSG samples were then used to set gating criteria for patINSG nuclei, which, under DMSO treatment, were largely GFP negative. In contrast, (*S*)-PHA533533 treatment of patINSG neurons resulted in a clear increase in GFP-positive nuclei, approaching levels seen in untreated matINSG cultures (Figure 11, A and B). To directly compare performance with the *Ube3a*-YFP model, we performed the same assay using nuclei isolated from patYFP neurons. In this model, (*S*)-PHA533533 induced a barely detectable shift in fluorescence intensity (Figure 11, C and D), underscoring the limitations of the diffuse YFP signal for nuclear-sorting-based analyses. Together, these results demonstrate that Sun1-GFP provides a robust, allele-specific nuclear signal suitable for flow cytometric detection and sorting, underscoring a key advantage of the *Ube3a*-INSG reporter over the *Ube3a*-YFP model.

**Figure 12:**
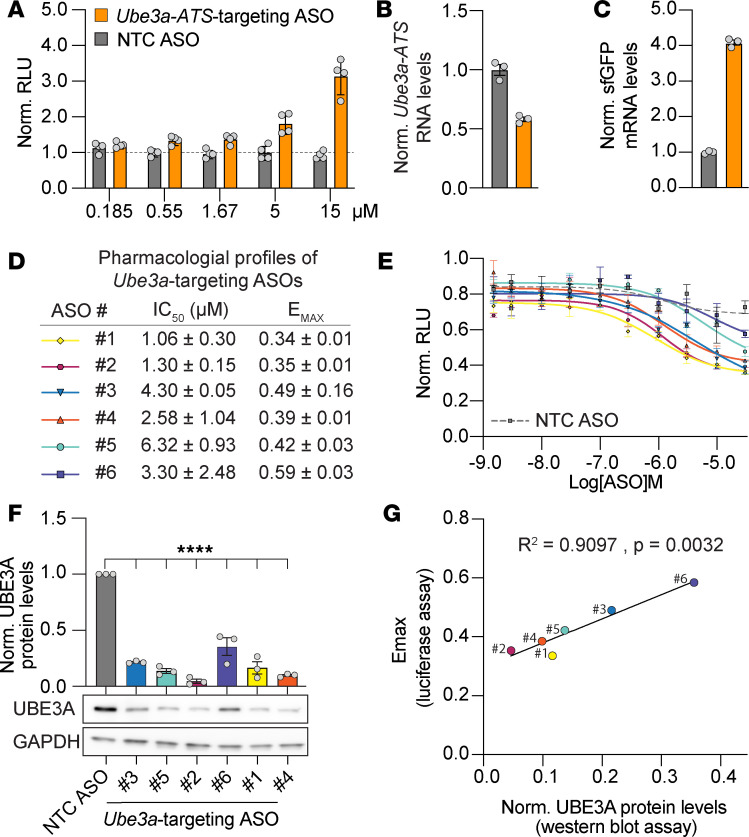
*Ube3a-ATS*–targeting and *Ube3a*-targeting ASOs respectively induce paternal *Ube3a* unsilencing or UBE3A downregulation in INSG neurons. (**A**–**C**) *Ube3a-ATS*–targeting ASO treatment in patINSG neurons showed dose-dependent paternal *Ube3a* unsilencing compared with non-targeting ASO, measured by luciferase assay (**A**), and by RT-qPCR at 5 μM ASO for *Ube3a-ATS* RNA knockdown (**B**) and sfGFP mRNA upregulation (**C**) normalized to *Eif4a2* (*N* = 3–4 wells per group). (**D** and **E**) Summary of pharmacological profiles (IC_50_ and E_max_) from luciferase-based *Ube3a* knockdown assays in matINSG neurons by *Ube3a*-targeting ASOs (**D**) with representative dose-response curves versus non-targeting control (NTC) ASO (**E**) (*N* = 3 runs in quadruplicate). (**F**) UBE3A protein levels quantified by Western blot in WT neurons treated with 10 μM NTC or *Ube3a*-targeting ASOs (*N* = 3). (**G**) Correlation between E_max_ values from luciferase assay and UBE3A protein levels for *Ube3a*-targeting ASOs, with Pearson’s *R*^2^ and *P* value indicated. ASO treatments lasted 72 hours. Data are represented as mean ± SEM. Statistical analysis by 1-way ANOVA with Bonferroni’s post hoc (**F**) and 2-tailed *t* tests (**G**). ****P < 0.0001. Norm., normalized.

In addition to small-molecule reactivation, we assessed whether the INSG model could report antisense oligonucleotide–mediated (ASO-mediated) unsilencing of paternal *Ube3a* (Figure 12). To this end, we tested a previously validated ASO (ASO RTR26183) targeting *Ube3a-ATS* (13). Treatment of patINSG neurons with this ASO resulted in a dose-dependent increase in Nluc signal (Figure 12A), indicating paternal *Ube3a* unsilencing. This unsilencing was independently confirmed by reverse transcription quantitative PCR (RT-qPCR), which showed decreased *Ube3a-ATS* RNA together with increased sfGFP mRNA levels (Figure 12, B and C, respectively). These results indicate that INSG reporter fidelity is preserved across distinct assay modalities.

To evaluate the utility of the *Ube3a*-INSG model beyond paternal unsilencing, we applied it to screen therapeutic candidates aimed at downregulating *Ube3a*. Dup15q syndrome is a neurodevelopmental disorder caused by maternal duplication or triplication of the 15q11.2–q13.1 region, which includes multiple genes, including *UBE3A* (41). Because maternally inherited duplications lead to more severe phenotypes than paternal duplications (42–44), UBE3A overexpression specifically in neurons is thought to be a major contributor to Dup15q syndrome (45–48). Accordingly, reducing UBE3A levels represents a promising therapeutic strategy. To this end, we screened 6 *Ube3a*-targeting ASOs in matINSG neurons. ASO treatment resulted in dose-dependent reductions in Nluc signal (Figure 12, D and E). Importantly, maximum effect (E_max_) values from the luciferase assay correlated with UBE3A protein knockdown, as measured by Western blot, in WT neurons (Figure 12, F and G), validating the reporter as a proxy for endogenous protein levels. Together, these results highlight the value of the Nluc reporter for efficiently screening therapeutic compounds and ASOs in primary neuron cultures to identify drug/ASO candidates for both AS and Dup15q syndromes.

### Nluc reporter enables in vivo bioluminescence imaging.

**Figure 13:**
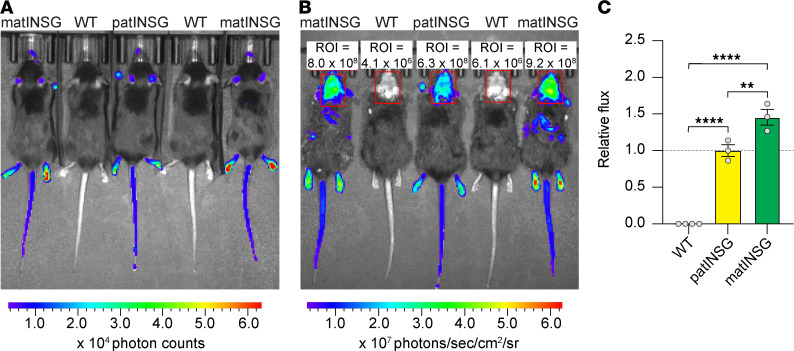
INSG mice enable in vivo quantification of UBE3A levels by bioluminescence imaging. (**A**) Representative bioluminescence signal from 12- to 13-week-old adult female matINSG, patINSG, and WT mice following systemic administration of the Nluc substrate fluorofurimazine. (**B**) Representative images from the same animals after scalp hair removal and repeat substrate administration. (**C**) Quantification of total radiance from matINSG, patINSG, and WT mice, measured as the integrated signal within a standardized head region of interest (ROI) applied uniformly across animals, as indicated. Data were normalized to patINSG mice within the experiment and analyzed by 1-way ANOVA with Bonferroni’s post hoc test. Individual points represent single animals (*N* = 3–4 per group). Data are shown as mean ± SEM. **P < 0.005, ****P < 0.0001. sr, steradian.

Finally, we assessed whether the Nluc reporter allows in vivo bioluminescence imaging (BLI) in the INSG system. BLI offers a highly sensitive, noninvasive, and non-terminal method for pharmacodynamic profiling, making it well suited for preclinical therapeutic studies. To evaluate this, 12- to 13-week-old adult female matINSG, patINSG, and WT littermates were injected with the fluorofurimazine substrate and imaged using an IVIS Spectrum system (Figure 13). Consistent with known UBE3A expression in peripheral tissues such as skin, we observed strong Nluc bioluminescent signal from the paws, ears, nose, and tail of matINSG and patINSG mice, whereas WT mice showed no detectable signal (Figure 13A). These regions are naturally free of black fur in C57BL/6J mice, which enhances detection and underscores why albino/nude strains are commonly used for in vivo BLI (49).

To assess cranial signals, presumably reflecting reporter expression in the brain and overlaying tissues, we chemically removed scalp hair and repeated imaging 1 week later. As expected, signal from the tail and paws persisted, but importantly, bioluminescence from the scalp region became detectable in both matINSG and patINSG mice (Figure 13B). Although patINSG mice showed a robust cranial signal likely arising from non-neuronal sources and complicated by skin UBE3A expression, quantitative analysis revealed a clear increase in cranial signal intensity in matINSG mice, with approximately 50% higher signal in comparison with patINSG animals (Figure 13C). These results demonstrate that the INSG model enables in vivo BLI for noninvasive, quantitative assessment of paternal *Ube3a* unsilencing, as reflected by higher signals in matINSG compared with patINSG mice.

## Discussion

We developed the *Ube3a*-INSG mouse model to accelerate preclinical development of therapeutics for AS. *Ube3a*-INSG integrates 2 complementary reporters: Nluc and Sun1-sfGFP. Nluc enables high-throughput, quantitative measurement of UBE3A expression and noninvasive BLI in vivo. Sun1-sfGFP, a nuclear envelope–localized GFP fusion, facilitates flow cytometry and single-cell monitoring of UBE3A expression throughout the brain. This dual-reporter design introduces new capabilities that streamline therapeutic development and provides flexibility to use the same model across the entire preclinical pipeline — from rapid in vitro compound screening to detailed in vivo assessment of biodistribution and pharmacodynamic effects.

Because paternal *Ube3a* silencing is neuron specific, unsilencing must be evaluated in postmitotic neuronal systems, historically limiting the scalability of screening. To date, only about 5,100 small molecules have been screened across 2 published studies using high-content assays to identify paternal *Ube3a* unsilencers (9, 10). The *Ube3a*-INSG model enables far more scalable and rapid luciferase-based screening in primary neurons, supporting additional small-molecule screening efforts. We demonstrated this screening capability by successfully replicating the pharmacological profile of (*S*)-PHA533533, a known *Ube3a* unsilencer, using the INSG model (10). Our luciferase assay yielded comparable potency and toxicity data to those from ICC-based detection in the *Ube3a*-YFP model, but at significantly reduced time, cost, and technical complexity. Furthermore, we demonstrated the broad utility of the Nluc reporter by dose-dependent increases in luciferase signal in patINSG neurons following treatment with a *UBE3A-ATS*–targeting ASO, validating the model for high-throughput screening of both small molecules and ASO-based *Ube3a* unsilencers.

Beyond identifying unsilencers, the Nluc readout in matINSG neurons enables screening of compounds that reduce UBE3A levels, an application directly relevant to Dup15q syndrome. In Dup15q, maternal duplications or triplications of the 15q11.2–q13.1 region, where *UBE3A* resides, lead to its overexpression, which contributes to core symptoms such as autism spectrum disorder, intellectual disability, and epilepsy (41). *Drosophila melanogaster* Dup15q models, in which overexpression of UBE3A homolog Dube3a induces spontaneous and pharmacoresistant seizures, have provided valuable insights into glial contributions to hyperexcitability and enabled high-throughput identification of seizure-suppressing compounds (50–53). However, no current preclinical mouse model offers comparable throughput for large-scale screens of UBE3A-lowering agents. Therefore, the *Ube3a*-INSG reporter and *Drosophila* seizure models are highly complementary, as UBE3A-lowering candidates identified using the INSG platform can subsequently be evaluated in seizure models to assess their functional impact. Thus, the Nluc-based readout enables scalable screening for UBE3A-lowering compounds in matINSG neurons and *Ube3a* unsilencers in patINSG neurons, supporting therapeutic discovery in both AS and Dup15q syndrome.

The Nluc reporter in the INSG model also supports noninvasive in vivo bioluminescence imaging. Systemic fluorofurimazine administration produced high cranial luminescence in matINSG mice, with lower levels observed in patINSG cranial areas. This approach could enable longitudinal assessment of UBE3A reporter activity in the same animals, providing a practical tool for tracking pharmacodynamic responses over time. Such repeated measures designs enhance statistical power and allow evaluation of drug onset, duration, and efficacy without the need for serial tissue collection.

The strategic localization of Sun1-sfGFP to the nuclear envelope enables 3 key capabilities not achievable with prior models: allele-specific nuclear quantification of UBE3A in individual cells by IHC and flow cytometry and whole-brain cellular profiling using LSFM. The sharp, ring-like signal of Sun1-sfGFP provides clear resolution of individual cells for microscopy. This unambiguous morphology is amenable to automated segmentation pipelines such as InstanSeg, facilitating quantification of UBE3A expression at single-cell resolution (54). When combined with cell type–specific markers, this system allows precise analysis within defined cellular populations, which we demonstrated in the CA1 hippocampal field. Such unsupervised, high-resolution quantification is impractical with the diffuse signal of UBE3A-YFP or UBE3A immunolabeling, especially in densely packed brain regions (22). Consistent with this, the INSG model is fully compatible with flow cytometry. We observed a clear increase in GFP-positive nuclei following (*S*)-PHA533533 treatment of patINSG neurons, validating Sun1-sfGFP as a robust allele-specific reporter for detecting UBE3A expression at the cellular level. This capability enables powerful downstream applications, including nuclear sorting for genetic and transcriptional interrogation of *Ube3a* unsilencing.

Sun1-sfGFP also enables whole-brain imaging with LSFM, which we used to visualize UBE3A expression across intact brains at cellular resolution. This is particularly valuable for AS therapeutic development, where treatment efficacy depends not only on restoring UBE3A levels but also on achieving a widespread and uniform distribution of interventions. Conventional IHC-based methods to evaluate therapeutic coverage are labor-intensive and spatially limited. In contrast, LSFM supports rapid, brain-wide imaging of cleared tissues at single-cell resolution. When combined with computational toolkits like MIRACL and ClearMap, this approach enables automated alignment to brain atlases and region-specific quantification, including cellular-level analysis in regions with low to moderate cell density, such as the neocortex (55, 56). These capabilities allow precise mapping of therapeutic distribution, identification of under-targeted areas, and evaluation of dose-dependent effects, providing a scalable and robust framework for optimizing delivery strategies. As such, Sun1-sfGFP combined with LSFM and advanced image analysis substantially improves the precision and efficiency of in vivo assessment in preclinical AS studies.

While the *Ube3a*-INSG model presents clear advantages over historical reporter models, some limitations should be noted. Though UBE3A protein levels appear unaffected, the insertion of the reporter cassette into the *Ube3a* 3′-UTR results in reduced transcript levels in both matINSG and patINSG brains. This suggests potential effects on transcript stability or processing that warrant caution, particularly in studies focused on mRNA dynamics. Although biochemical and histological assessments indicate no major abnormalities, it is impossible to fully exclude subtle molecular or physiological effects caused by the knockin construct. Therefore, we conducted anatomical and behavioral assessments, which are sensitive to UBE3A loss- and gain-of-function effects (27, 30, 57). The absence of abnormalities provides strong in vivo evidence that UBE3A protein function is preserved in the INSG model. Future detailed molecular characterizations of transcript dynamics would be valuable for a comprehensive understanding of the potential impacts of the reporter insertion. Additionally, the 2 reporters (Nluc and Sun1-sfGFP) differ in expression kinetics and stability from endogenous UBE3A, which may cause temporal mismatches when detecting rapid or transient changes, particularly relevant for short-term pharmacological studies requiring precise timing.

In summary, the *Ube3a*-INSG mouse model offers a comprehensive and versatile tool for advancing therapeutic development for AS and Dup15q syndrome. Its dual-reporter design enables high-throughput compound screening, single-cell quantification, and whole-brain evaluation. These integrated capabilities position the *Ube3a*-INSG model as a powerful platform to accelerate preclinical research and support the discovery of safe and effective therapies for AS and other UBE3A-related disorders.

## Methods

### Sex as a biological variable

Both male and female animals were included. Behavioral outcomes were comparable across sexes, and data were pooled, whereas body weight was analyzed by sex. For primary neuron cultures, cortices from embryos of both sexes were pooled within litters.

### CRISPR/Cas9 reagents

Guide RNAs (gRNAs) targeting the mouse *Ube3a* 12th intron and 3′-UTR (60–80 bp from the stop codon) were designed using Benchling (RRID:SCR_013955). To minimize off-target effects, high-fidelity eSpCas9 was used together with transfer RNA (tRNA)–gRNA fusion RNAs to avoid heterologous 5′ nucleotide additions that impair Cas9 activity. gRNAs were cloned into a T7-tRNA-gRNA construct (University of North Carolina [UNC] Animal Models Core), transcribed in vitro using the HiScribe T7 High Yield RNA Synthesis Kit (E2040S, New England Biolabs), and purified via RNeasy spin columns (74104, QIAGEN) in microinjection buffer (5 mM Tris-HCl [pH 7.5], 0.1 mM EDTA). gRNA activity was validated by electroporation of each gRNA with recombinant eSpCas9 into mouse embryonic fibroblasts (UNC Animal Models Core). Cells were harvested about 72 hours after electroporation, and target sites were PCR-amplified, sequenced, and analyzed using the Inference of CRISPR Edits (ICE, Synthego Inc., RRID:SCR_024508). The selected gRNAs were Ube3a-5g62B (5′-ATGTATATCGAAGTCTACCT-3′) and Ube3a-3g41T (5′-ATATAAGAGGGATAATTTGA-3′). A donor plasmid was cloned with the following elements: (a) 1,002 bp 5′ homology arm (upstream of the Ube3a-5g62B protospacer adjacent motif [PAM], with the PAM mutated from AGG to AGC); (b) 669 bp WT sequence spanning from the mutated PAM in intron 12 to the *Ube3a* stop codon in exon 13; (c) 3,566-bp INSG reporter cassette containing IRES, Nluc, 2A self-cleaving peptide from *Thosea asigna* virus capsid protein (T2A), Sun1 nuclear envelope localization domain fused to the N-terminus of superfolder GFP (Addgene 160141), and FRT site; (d) 63 bp WT sequence from the start of the *Ube3a* 3′-UTR to the Ube3a-3g41T PAM (with the PAM mutated from TGG to TTG); and (e) 997 bp 3′ homology arm immediately downstream of the mutated PAM. The donor vector was prepared using a High Speed Maxiprep (12662, QIAGEN), eluted in microinjection buffer, and dialyzed using a 0.05 μM mixed cellulose ester membrane (VMWP02500, Millipore) against a 20-fold excess of microinjection buffer for at least 1 hour.

### Embryo microinjection

C57BL/6J zygotes were microinjected with a mixture containing 400 nM eSpCas9 protein, 33 ng/μL of each gRNA, and 20 ng/μL donor plasmid in microinjection buffer (5 mM Tris-HCl [pH 7.5], 0.1 mM EDTA) and then implanted into pseudopregnant B6D2F1 females. Founder pups were screened by PCR for correct INSG cassette integration at the 5′ and 3′ junctions. One female founder carrying the intended insertion, but also exhibiting vector backbone sequences, was crossed with a Flp-transgenic male C57BL/6J-Tg(CAG-Flpo) (UNC Animal Models Core) to eliminate tandem integrations. One F_1_ male with a correct single-copy insertion and lacking the Flp transgene was bred to WT C57BL/6J females to establish the line.

### Mice

Knockin mice expressing yellow fluorescent protein (YFP) fused to the C-terminus of *Ube3a* were originally generated in the laboratory of A. Beaudet (Baylor College of Medicine, Houston, Texas, USA) (21) (RRID:IMSR_JAX:017765). Both *Ube3a*-YFP and *Ube3a*-INSG mice were maintained on a coisogenic C57BL/6J background under standard housing conditions with ad libitum access to food and water. Paternal or maternal inheritance of the *Ube3a* reporter allele was generated by crossing of WT females with reporter males or of WT males with reporter females, respectively. Genotyping primers are listed in Supplemental Table 1.

### Western blot analysis

Tissues were snap-frozen in liquid nitrogen and stored at –80°C until lysis. Primary mouse neurons were washed once with phosphate-buffered saline (PBS) before lysis. Cells and tissues were lysed in ice-cold RIPA lysis buffer (50 mM Tris-HCl [pH 8.0], 150 mM NaCl, 1% NP-40, 0.5% sodium deoxycholate, 0.5% SDS) supplemented with protease inhibitor cocktail (P8340, MilliporeSigma). Cultured cells were lysed by repeated pipetting with a 1 mL pipette tip in lysis buffer, while tissues were homogenized using a Tissue Tearor (model 985-370, BioSpec). Lysates were incubated on ice for 10 minutes, sonicated 3 times for 5 seconds, and centrifuged at maximum speed for 10 minutes at 4°C to remove insoluble cell debris. Protein concentrations were measured using the Pierce BCA protein assay kit (23227, Thermo Fisher Scientific). A total of 30 μg of each sample was separated on 7.5% Mini-PROTEAN TGX gel (4561024, Bio-Rad) and transferred at 90 mA for 90 minutes onto 0.45 μm Immobilon-FL PVDF membrane (05317, MilliporeSigma) in ice-cold transfer buffer (25 mM Tris-base, 192 mM glycine, and 20% MeOH). Membranes were blocked in Intercept (PBS) Blocking Buffer (927-70001, LI-COR Biosciences) for 1 hour and incubated with primary antibodies (Supplemental Table 2) overnight at 4°C. After washing 3 times with PBS/0.5% Tween 20, membranes were incubated with HRP-conjugated secondary antibodies for 1 hour at room temperature. Chemiluminescence signals were detected using Clarity Western ECL substrate (1705061, Bio-Rad), imaged on an Amersham Imager 680 (GE Healthcare), and quantified using ImageJ (RRID:SCR_003070).

### RNA isolation and RT-qPCR

For tissue RNA extraction, half-brains from adult mice were snap-frozen immediately after dissection and stored at –80°C until processing. Tissues were lysed in RLT buffer (79216, QIAGEN) with 1% β-mercaptoethanol, and total RNA was isolated using the RNeasy Mini kit (74104, QIAGEN) according to the manufacturer’s protocol. For cultured neurons, media were removed, cells were washed once with PBS, and RNA was isolated using TRIzol reagent (250 μL per well; 15596026, Thermo Fisher Scientific) following the manufacturer’s protocol. cDNA was synthesized from 1 μg (tissue) or 250 ng (neurons) of total RNA using qScript cDNA Supermix (95048, Quantabio). RT-qPCR reactions were performed using one-twenty-fifth of the synthesized cDNA with PowerUp SYBR Green Master Mix (A25742, Applied Biosystems) and gene-specific primers (Supplemental Table 3) on a QuantStudio 5 Real-Time PCR system (Applied Biosystems) with melting curve analysis to confirm specificity. *Ube3a* primers were obtained from ref. 58. Assays were performed in technical quadruplicates, averaged per sample, and normalized to *Eif4a2* using the comparative Ct (ΔΔCt) method.

### Tissue preparation for histology

Mice received intraperitoneal injections of Euthasol (a mixture of pentobarbital sodium and phenytoin sodium, 100 mg/kg, i.p.; ANADA 200-071, Virbac) for deep anesthesia, followed by intracardiac perfusion with PBS (0.1 M, pH 7.3) and then with 4% freshly depolymerized paraformaldehyde in phosphate buffer (pH 7.4) for 10 minutes. Brains were extracted, postfixed overnight at 4°C in the same fixative, and cryoprotected in 30% sucrose in PBS until saturated. For confocal and stimulated emission depletion (STED) microscopy, brains were sectioned at 50 μm thickness using a sliding microtome and stored at –20°C in a cryopreservative solution (45% PBS, 30% ethylene glycol, 25% glycerol) until use.

### Immunolabeling for confocal and STED microscopy

Free-floating sections were rinsed twice in PBS (5 minutes each), permeabilized in PBS containing 0.1% Triton X-100 (PBS-T), and blocked for 30 minutes at room temperature in PBS-T containing 10% FBS. Sections were incubated overnight with primary antibodies (Supplemental Table 2), followed by PBS-T washes and overnight incubation with fluorophore-conjugated secondary antibodies. Sections were counterstained with DAPI (1 μg/mL; D1306, Invitrogen), washed, and mounted on gelatin-coated slides. Slides were air-dried and coverslipped using Vectashield Plus (H-1900, Vector Laboratories) for confocal microscopy or Abberior Mount Liquid (MM-2007, Abberior GmbH) for STED imaging. Images were acquired using a Leica STELLARIS 8 FALCON microscope and quantified using QuPath (RRID:SCR_018257; ref. 59) and InstanSeg (https://github.com/instanseg; ref. 54). InstanSeg uses a deep learning convolutional neural network to enable accurate segmentation of cellular and nuclear boundaries for single-cell quantification.

### Immunolabeling for LSFM

Brain clearing and immunolabeling followed the immunolabeling-enabled 3-dimensional imaging of solvent-cleared organs (iDISCO) protocol (38). Briefly, brains were sequentially dehydrated in methanol/PBS (5%–100%, 30 minutes per step), incubated overnight in 66% dichloromethane/33% methanol, washed twice in 100% methanol, and bleached in 5% H_2_O_2_ in methanol at 4°C for 24 hours. Tissues were rehydrated through a descending methanol series (100%–10%, 30 minutes per step), washed twice in 0.2% Triton X-100/PBS for 1 hour, and permeabilized at 37°C for 48 hours (0.2% Triton X-100, 2% glycine, 20% DMSO in PBS). Tissues were blocked for 48 hours (0.2% Triton X-100, 1% BSA) and incubated with primary antibodies (Supplemental Table 2) for 6 days in antibody solution (0.2% Tween 20, 40 mg/L heparin, 11% BSA, 5% DMSO in PBS). After washing (0.2% Tween 20, 40 mg/L heparin in PBS), samples were incubated with secondary antibodies for 6 days, followed by dehydration in methanol, dichloromethane treatment, and clearing in dibenzyl ether. Imaging was performed using Ultramicroscope II (LaVision BioTec) and visualized with Imaris (RRID:SCR_007370).

### Behavioral assays

Behavioral testing began at about P70. Assays were administered in order of increasing stress, with 2–3 days between tests.

#### Open field.

Locomotor activity in a novel environment was assessed for 30 minutes in an open field chamber (40 cm × 40 cm × 30 cm) contained inside sound-attenuating boxes with ceiling-mounted lights. Mice were video-recorded, and total distance traveled was quantified in 5-minute bins using Ethovision XT 15.0 (Noldus, RRID:SCR_000441).

#### Marble burying.

Mice were placed for 30 minutes in clean cages containing 3 L of corncob bedding (~5 cm deep; Andersons Lab; 1/8 inch in diameter, irradiated) with 20 black glass marbles (14 mm diameter) arranged in a 5 × 4 grid. Overhead images were acquired before and after testing, and the percentage of marble area obscured by bedding was quantified using ImageJ (NIH; RRID:SCR_003070).

#### Rotarod.

Balance and motor coordination were assessed on an accelerating rotarod (Ugo Basile). Mice were tested from 4 to 40 rpm over 5 minutes. Three trials were conducted during the initial session and 2 additional trials 48 hours later, with 3- to 5-minute intertrial intervals. Latency to fall or to 3 consecutive passive rotations was recorded.

#### Nest building.

Mice were single-housed for 3 days before testing. Nesting material was replaced with 11 ± 1 g of compressed blot filter paper (1703966, Bio-Rad) cut into 8 equal rectangles, and unused material was weighed daily for 5 days.

### Drug and ASO preparation

(*S*)-PHA533533 was synthesized as previously described (37, 60, 61). Octanoic acid was purchased from Sigma-Aldrich (O3907, lot SHBL4414). For in vitro assays, all drugs were formulated in DMSO (0.1% final). For in vivo assays, (*S*)-PHA533533 was dissolved in 0.9% NaCl. All ASOs were synthesized by Integrated DNA Technologies and diluted in PBS (Supplemental Table 4). The *Ube3a-ATS*–targeting ASO (named RTR26183), previously described (13), is a fully phosphorothioate-modified oligonucleotide composed of locked nucleic acid (LNA) and DNA bases, with 5-methylcytosine–modified LNA cytosines. The 6 *Ube3a*-targeting ASOs (ASOs 1–6), described in patent WO2023239782A2 (62), are 2′-*O*-methoxyethyl gapmers with a phosphorothioate backbone.

### Mouse primary neuron cultures

Timed pregnant female mice (3–5 months old) were euthanized via cervical dislocation. Cortices from E15.5 embryos from both sexes were dissected in Leibovitz’s L-15 Medium (11415064, Thermo Fisher Scientific) and rinsed with HBSS (14025076, Thermo Fisher Scientific). Cortices were then incubated for 30 minutes at 37°C in papain (250 μL per brain; 1 vial diluted in 2.5 mL HBSS; 88285, Thermo Fisher Scientific) with DNase I (20 mg/mL; D4513, MilliporeSigma). To deactivate the papain, 1 mL of DMEM containing 10% FBS (TMS-013-B, MilliporeSigma), GlutaMax (35050-061, Invitrogen), and Antibiotic-Antimycotic (15240-062, Invitrogen) was added to the cortical tissue, followed by trituration. The cells were centrifuged for 2 minutes at 4,600*g*, washed with HBSS, and resuspended in DMEM with 10% FBS, GlutaMax, and Antibiotic-Antimycotic. The cells were filtered through a 70 μm filter and incubated at 37°C for 15 minutes on a sterile tissue culture plate to remove glial cells (similarly to the method described in ref. 63). The supernatant was carefully collected, and cells were plated in Neurobasal Plus Medium (A3582901, Thermo Fisher Scientific) containing B27 Plus (A3582801, Thermo Fisher Scientific), GlutaMax (35050-061, Invitrogen), and Antibiotic-Antimycotic (15240-062, Invitrogen) onto poly-d-lysine–coated, black-walled, 384-well plates at 2 × 10^4^ cells per well for dose-response assays, 24-well plates at 2.5 × 10^5^ cells per well for RT-qPCR assays, or 6-well plates at 1.5 × 10^6^ cells per well for flow cytometry. Cultured neurons were maintained by half-medium changes every 3–4 days using Neurobasal Plus, GlutaMax, B27 Plus, and 2.46 μg/mL 5-fluoro-2′-deoxyuridine (F0503, MilliporeSigma).

### ICC, high-content imaging, and dose-response analysis in cultured neurons

The immunofluorescence protocol for high-content imaging was previously described (10). Neurons were treated at 7 days in vitro (DIV7) with (*S*)-PHA533533 or 0.1% DMSO for 72 hours, washed with PBS, and fixed in 4% paraformaldehyde for 10 minutes at room temperature. Cells were permeabilized with 1% Triton X-100 in PBS for 10 minutes and blocked for 30 minutes in PBS containing 5% normal growth serum and 0.2% Triton X-100 (NGST). Neurons were incubated overnight at 4°C with primary antibodies in NGST (Supplemental Table 2), followed by incubation with secondary antibodies and DAPI (133 ng/mL; D1306, Invitrogen) for 60 minutes at room temperature. Images were acquired on a Nikon Ti2 Eclipse fluorescence microscope (Nikon Instruments) and analyzed using NIS-Elements software. Potency (EC_50_) of paternal *Ube3a*-INSG unsilencing was determined using 2 complementary ICC-based metrics: (a) mean normalized GFP intensity in DAPI^+^NeuN^+^ neurons relative to DMSO controls and (b) the percentage of GFP^+^DAPI^+^NeuN^+^ neurons, using a GFP threshold set to yield approximately 5% GFP^+^ neurons in DMSO-treated wells. Cytotoxicity (CC_50_) was assessed as the percentage of surviving DAPI^+^NeuN^+^ neurons relative to DMSO controls. For E_max_ determination, only concentrations resulting in less than 10% reduction in neuronal survival were included; the highest concentration within this nontoxic range defining the response plateau was designated as the maximal effect.

### Luciferase assay and dose-response analysis

For the dose-response assay for (*S*)-PHA533533 (Figure 8, C and D) and *Ube3a*-targeting ASOs (Figure 8, I and J), neurons were treated at DIV7 for 72 hours. Cells were washed with PBS and lysed with 1× Passive Lysis Buffer (25 μL/well; E1941, Promega) for 15 minutes. Nluc was measured using coelenterazine H (301, NanoLight Technology), prepared as a 1 mM stock in ethanol and diluted to 20 μM in ultrapure Milli-Q water, with luminescence recorded over a 1-second integration time on a CLARIOstar Plus (BMG Labtech) microplate reader with injector (10 μL substrate per well). Relative light units were normalized to DMSO-treated controls. EC_50_ values for patINSG unsilencing were determined using concentrations that produced increasing luminescence up to a plateau, whereas CC_50_ values were calculated from concentrations beyond the plateau where luminescence declined as a result of cell loss.

For dose-response assays of *Ube3a*-targeting ASO (Figure 8F) and octanoic acid together with (*S*)-PHA533533 (Supplemental Figure 2, A and B), neurons were treated at DIV7 for 72 hours. Cell viability was assessed using CellTiter-Fluor assay (G6080, Promega) in 25 μL of fresh medium containing 20% of assay buffer and 25 μM of GF-AFC substrate per well, followed by luminescence measurement using the Nano-Glo Luciferase Assay System (N1110, Promega), according to the manufacturers’ protocols. Fluorescence and luminescence were measured on a CLARIOstar Plus (BMG Labtech) microplate reader.

### Isolation of nuclei and flow cytometry analysis

Neuronal cultures were maintained for 10 days in 6-well plates at 1.5 × 10^6^ neurons per well. Nuclei were isolated by lysing of each well in 0.5 mL Nuclei PURE lysis buffer (NUC102, Sigma-Aldrich) supplemented with 0.5 μL 1 M DTT and 5 μL 10% Triton X-100. Lysates were collected, triturated, incubated on ice for 5 minutes, and centrifuged at 500*g* for 5 minutes. Pellets were resuspended in 250 μL cold DPBS containing 1% BSA (SP-5050-500, Vector Laboratories), mixed with 400 μL 2 M sucrose (S9308, Sigma-Aldrich), layered onto 250 μL 2 M sucrose, and centrifuged at 13,000*g* for 45 minutes to separate nuclei from debris. Nuclei were resuspended in 500 μL DPBS containing 1% BSA and 2 μg/mL DAPI (D1306, Invitrogen) (adapted from ref. 64).

Flow cytometry was performed on a FACSMelody cell sorter (BD Biosciences) with a 100 μm nozzle. A total of 10,000 nuclei events were acquired per replicate. Nuclei were gated by DAPI fluorescence with violet laser, followed by FSC-A/SSC-A to select low-complexity events; GFP and YFP were excited with the blue laser. Data were analyzed using FlowJo v10.10 (BD Biosciences; RRID:SCR_008520).

### In vivo BLI

Female 12- to 13-week-old matINSG, patINSG, and WT littermate mice were used for in vivo BLI. Mice were briefly anesthetized with 2.5% isoflurane and injected intraperitoneally with fluorofurimazine (FFz; 2 mg/kg; 50-313-4464, Selleck Chemicals) dissolved in 10% DMSO, 40% PEG300, and 50% DPBS. After a 5-minute recovery, mice were re-anesthetized for imaging, with anesthesia maintained at 1.5% isoflurane and the stage temperature set to 37°C. Images were acquired at 10 minutes after FFz administration using an IVIS Spectrum system (Revvity Inc.) with the following settings: field of view D, focus 1.5 cm, binning 8, f/stop 1, and 5-second exposure (no emission filter). One week later, the same animals were imaged under identical conditions after chemical hair removal over the skull using depilatory cream. Background signal was determined from WT mice, subtracted with clipping, and normalized to patINSG mice within each imaging session.

### Statistics

All data analyses were conducted using GraphPad Prism 10.4.1 (RRID:SCR_002798), except for Figure 7, which was analyzed using OriginPro 2023b (OriginLab). All individual data values, statistical tests, sample sizes (*N*), and significance thresholds are provided in the Supporting Data Values file and referenced in the main text or figure legends. A *P* value less than 0.05 was considered statistically significant.

### Study approval

All animal studies were conducted in accordance with NIH guidelines under an IACUC-approved protocol at the University of North Carolina School of Medicine.

### Data availability

All data supporting the findings of this study are provided in the Supporting Data Values file. Full-resolution light-sheet microscopy datasets are available upon reasonable request and can be shared via an external hard drive or institutional file transfer services. No custom code was generated for this study; all analyses were performed using commercially available software as described in Methods.

## Author contributions

HV and DOC designed the *Ube3a*-INSG reporter mouse. ALS performed in vivo dosing. HV, ALS, HCN, and SL established primary neuronal cultures. ALS and LMJ designed, conducted, and analyzed behavioral experiments. ALS and SL performed Western blotting. LMJ and SL conducted RT-qPCR analysis. LMJ performed in vitro ASO assays. HCN performed flow cytometry experiments and analysis, as well as octanoic acid assays. CAF and ACB performed histology and microscopy. ACB performed quantitative analysis of in vivo fluorescence. TV assisted with colony management and perfusions. HV conducted in vitro ICC, high-content analysis, and luciferase assays. LMJ and LX performed in vivo bioluminescence imaging and analysis. HV, LMJ, HCN, and ACB analyzed data and provided scientific supervision. HV, LMJ, ACB, and BDP oversaw the project. HV wrote the manuscript with input from all authors. BDP secured funding and provided intellectual guidance for the project. All authors reviewed, edited, and approved the final version of the manuscript.

## Funding support

This work is the result of NIH funding, in whole or in part, and is subject to the NIH Public Access Policy. Through acceptance of this federal funding, the NIH has been given a right to make the work publicly available in PubMed Central.

- NIH National Institute of Neurological Disorders and Stroke (NINDS) grants R01NS131615 and R01NS129914 (to BDP).
- A grant from the Angelman Syndrome Foundation (to BDP).
- NIH Cancer Center Core Support Grant P30CA016086 to the UNC Lineberger Comprehensive Cancer Center.
- NIH grant 1S10OD030300 (for confocal microscopy).
- NIH grant P30CA016086 and the North Carolina Biotech Center Institutional Support Grant 2016-IDG-1016 (for light-sheet microscopy).
- NIH/NINDS Neuroscience Center Support Grant P30NS045892 and NIH National Institute of Child Health and Human Development Intellectual and Developmental Disabilities Research Center Grant P50HD103573 (for high-content imaging).
- NIH grant P30CA016086 to the UNC Animal Models Core Facility.

## Supplementary Material

Supplemental data

Unedited blot and gel images

Supporting data values

## Version 1 02/03/2026

In-Press Preview

## Version 2 03/09/2026

Electronic publication

## Version 3 03/10/2026

Missing supplemental data file posted
