# Supplementary material for: A dual-reporter mouse for therapeutic discovery in Angelman syndrome
Source: JCI Insight. 2026 Feb 3;11(5):e197028. doi: 10.1172/jci.insight.197028 (PMC13041673; doi:10.1172/jci.insight.197028)
Supplement: Supplemental data [file jciinsight-11-197028-s308.pdf]

Supplementary Data
(Supplementary Figures and Tables)

A dual-reporter mouse for therapeutic discovery in Angelman syndrome

Hanna Vihma[1], Lucas M. James[1], Hannah C. Nourie[1], Audrey L. Smith[1],

Siyuan Liang[1], Carlee A. Friar[1], Tasmai Vulli[1], Lei Xing[1], Dale O. Cowley[2,3],

Alain C. Burette[1,#], and Benjamin D. Philpot[1,4,#]

[1]Department of Cell Biology & Physiology, Neuroscience Center, University of North Carolina at Chapel Hill, NC, USA

[2]Animal Models Core, University of North Carolina at Chapel Hill, NC, USA

[3]Department of Genetics, University of North Carolina at Chapel Hill, NC, USA

[4]Carolina Institute for Developmental Disabilities, University of North Carolina at Chapel Hill, NC, USA

[#]Corresponding authors:

BDP: 7008C Mary Ellen Jones, 116 Manning Drive, Campus Box 7545, Chapel Hill, NC 27599, USA. Telephone: +1 919-966-0031, bphilpot@med.unc.edu

ACB: 7004 Mary Ellen Jones, 116 Manning Drive, Campus Box 7545, Chapel Hill, NC 27599, USA. Telephone: +1 919-966-0031, alain_burette@med.unc.edu

Conflict of Interest Statement: DOC is employed by, has equity ownership in, and serves on the board of directors of TransViragen, the company contracted by UNC-Chapel Hill to manage its Animal Models Core Facility. BDP is a consultant for Astellas Gene Therapies.

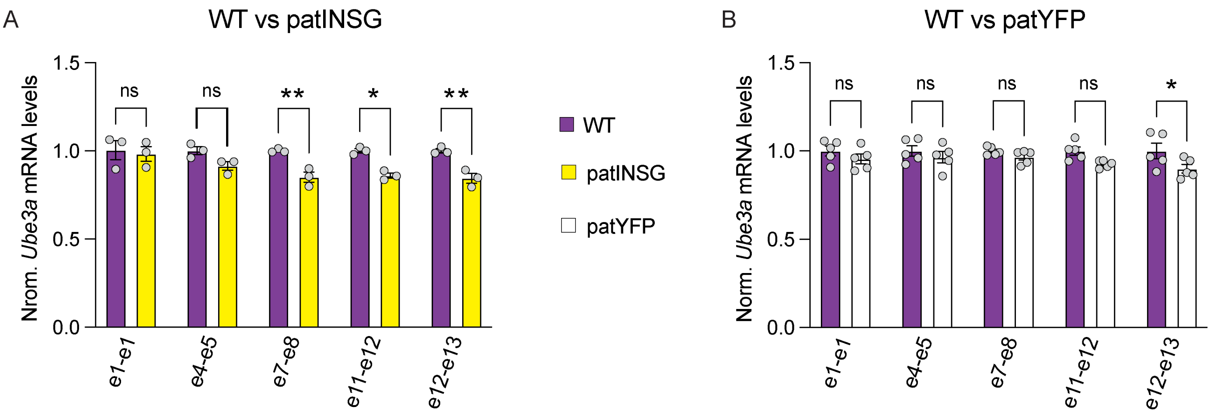

Supplementary Figure 1. Reporter insertion selectively reduces *Ube3a* mRNA levels toward the 3′ end of the transcript in both *Ube3a*-INSG and *Ube3a*-YFP mouse models. Quantification of *Ube3a* mRNA transcript levels in the brain by RT-qPCR using primer sets targeting different exons, as indicated in the figure, comparing (**A**) WT with patINSG and (**B**) WT with patYFP mice. Data are normalized to *Eif4a2* (two-way ANOVA, Bonferroni's *post hoc* test). Each data point represents an individual animal, with data shown as means ± SEM. ns = non-significant, *P < 0.05, **P < 0.005. Norm., normalized; e, exon.

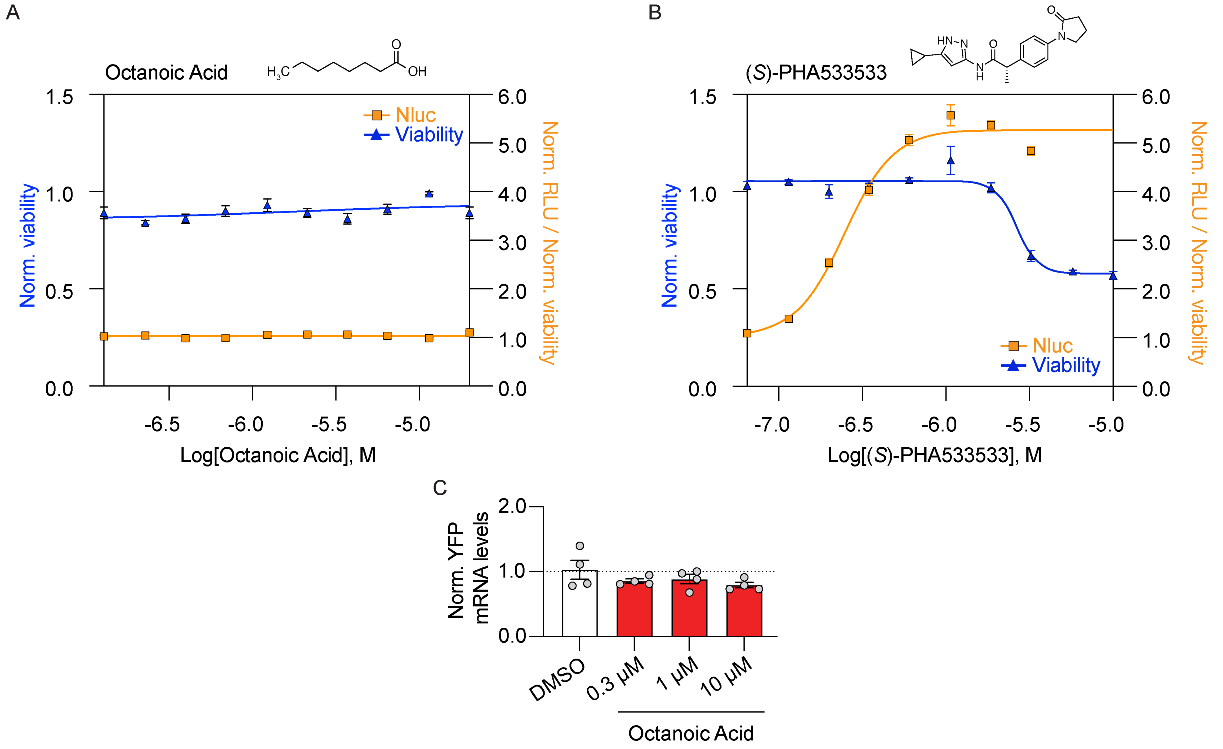

Supplementary Figure 2. Octanoic acid does not produce unsilencing of paternal *Ube3a* in patINSG or patYFP neurons. Dose response assay in patINSG neurons using luciferase readout normalized to viability for (**A**) octanoic acid, and (**B**) (*S*)-PHA533533, used as a positive control. (**C**) Quantification of YFP mRNA levels by RT-qPCR, normalized to *Eif4a2* levels, in patYFP neurons following octanoic acid treatments. Drug treatments were administered for 72 hours. Assays were performed as follows: (A-B) N = 1; performed in quadruplicate individual wells; (C) N = 1; performed in quadruplicate individual wells, each analyzed in quadruplicate. Norm, normalized.

Supplementary Table 1. Sequences of primers used for genotyping:

Mouse line	Primer name	Sequence
Ube3a-INSG	sfGFP_Forward	5’- GGA TCA CAT GAA ACG GCA TGA C -3’
	sfGFP_Reverse	5’- GTC TGC CGT GAT GTA TAC -3’
Ube3a-YFP	Ube3aYFP_Forward	5’- CAC ATG AAG CAG CAC GAC TT -3’
	Ube3aYFP_Reverse	5’- AGT TCA CCT TGA TGC CGT TC -3’

Supplementary Table 2. Antibodies used:

			Primary Antibody	Secondary Antibody
Confocal microscopy	GFP	Antibody	anti-GFP	Anti-chicken IgY, Alexa Fluor 488
		Host, type	Chicken, polyclonal	Donkey, polyclonal
		Dilution	1/1000	1/400
		Company, #Cat.	Abcam, ab13970	Jackson ImmunoResearch Labs, 703-545-155
		Reference	RRID:AB_300798	RRID:AB_2340375
	SOX9	Antibody	anti-SOX9	anti-goat IgG, Alexa Fluor Plus 555
		Host	Goat, polyclonal	Donkey, polyclonal
		Dilution	1/1000	1/400
		Company, #Cat.	R and D Systems, AF3075	Thermo Fisher Scientific, A32816
		Reference	RRID:AB_2194160	RRID:AB_2762839
	NeuN	Antibody	anti-NeuN	anti-guinea pig IgG, Alexa Fluor 594
		Host, type	Guinea pig, polyclonal	Donkey, polyclonal
		Dilution	1/1000	1/400
		Company, #Cat.	Millipore, ABN90	Jackson ImmunoResearch Labs, 706-585-148
		Reference	RRID:AB_11205592	RRID:AB_2340474
	UBE3A	Antibody	anti-UBE3A	anti-mouse IgG, Alexa Fluor 647
		Host, type	Mouse, monoclonal	Donkey, polyclonal
		Dilution	1/1000	1/400
		Company, #Cat.	Sigma-Aldrich, SAB1404508	Abcam, ab150111
		Reference	RRID:AB_10740376	RRID:AB_2890625
	DCX	Antibody	anti-doublecortin	Anti-rabbit pig IgG, Alexa Fluor 594
		Host, type	Rabbit, polyclonal	Donkey, polyclonal
		Dilution	1/1000	1/400
		Company, #Cat.	Cell Signaling, 4604S	Jackson ImmunoResearch Labs, 711-585-152
		Reference	RRID:AB_561007	RRID:AB_2340621
STED	GFP	Host, type	anti-GFP	anti-chicken IgY, Alexa Fluor 594
		Dilution	Chicken, polyclonal	Donkey, polyclonal
		Company, #Cat.	1/1000	1/400
		Reference	Abcam, ab13970	Jackson ImmunoResearch Labs, 703-585-155
		Reference	RRID:AB_300798	RRID:AB_2340377
	Lamin B1	Antibody	anti-Lamin B1	anti-rabbit IgG, ATTO 647N
		Host, type	Rabbit, polyclonal	Goat, polyclonal
		Dilution	1/1000	1/400
		Company, #Cat.	Abcam, ab16048	Rockland, 611-156-122
		Reference	RRID:AB_443298	RRID:AB_10893043
LSFM	GFP	Antibody	anti-GFP	anti-rabbit IgG, Alexa Fluor Plus 647
		Host, type	Rabbit, polyclonal	Donkey, polyclonal
		Dilution	1/1000	1/500
		Company, #Cat.	Novus, NB600-308	Thermo Fisher Scientific, A32795TR
		Reference	RRID:AB_10003058	RRID:AB 2866496
ICC	GFP	Antibody	anti-GFP	anti-rabbit IgG1, Alexa Fluor 488
		Host, type	Rabbit, polyclonal	Goat, polyclonal
		Dilution	1/1000	1/500
		Company, #Cat.	Novus, NB600-308	Thermo Fisher Scientific, A11008
		Reference	RRID:AB_10003058	RRID:AB 143165
	NeuN	Antibody	anti-NeuN	anti-mouse IgG, Alexa Fluor 568
		Host, type	Mouse, monoclonal	Goat, polyclonal
		Dilution	1/500	1/500
		Company, #Cat.	Millipore Sigma, MAB377	Thermo Fisher Scientific, A21124
		Reference	RRID:AB_2298772	RRID:AB 2535766
Western blotting	UBE3A	Antibody	anti-UBE3A	anti-mouse IgG, HRP
		Host, type	Mouse, monoclonal	Goat, polyclonal
		Dilution	1/1000	1/5000
		Company, #Cat.	Millipore Sigma, SAB1404508	Thermo Fisher Scientific, 31430
		Reference	RRID:AB_10740376	RRID:AB 228307
	GAPDH	Antibody	anti-GAPDH	anti-mouse IgG, HRP
		Host, type	Mouse, monoclonal	Goat, polyclonal
		Dilution	1/1000	1/5000
		Company, #Cat.	Millipore Sigma, MAB374	Thermo Fisher Scientific, 31430
		Reference	RRID:AB_2107445	RRID:AB 228307

Supplementary Table 3. Sequences of primers used for RT-qPCR:

Primer name	Sequence
mUbe3a_ex1_Forward	5'-TGGCGCCTCCTTCTGCTTCTCT-3'
mUbe3a_ex1_Reverse	5'-ACCTTGAAGTCGGCGCTGAAGC-3'
mUbe3a_e4_Forward	5'-ATCCCAGTCTGAGGACATTGA-3'
mUbe3a_e5_Reverse	5'-GCACAAAACTCATTCGTGCAG-3'
mUbe3a_e7_Forward	5'-GAGTAGATGAGGGAGGCGTT-3'
mUbe3a_e8_Reverse	5'-CAGACCCAGGACTATGCCAA-3'
mUbe3a_e11_Forward	5'-ACGGTGGCTATACGAGGGAA-3'
mUbe3a_e12_Reverse	5'-CCAACAGGTGCTCTGTCTGT-3'
mUbe3a_e12_Forward	5'-GCACCTGTTGGAGGACTAGG-3'
mUbe3a_e13_Reverse	5'-GTGATGGCCTTCAACAATCTC-3'
Eif4a2_Forward	5'-TCTCAATACAAGGCCCAAGG-3'
Eif4a2_Reverse	5'-CTCTTTCCTTCTGGTCCATGTC-3'
mUbe3a-ATS_Forward	5'-ACAGAACAATAGGTCACCAGGTT-3'
mUbe3a-ATS_Reverse	5'-AAGCAAGACTGTTCACCTCAT-3'
sfGFP_Forward	5'-TGTCCGTGGAGAGGGTGAAGGT-3'
sfGFP_Reverse	5'-GCACGCGTCTTGTAGGTCCCGT-3'
YFP_Forward	5'-ACATGAAGCAGCACGACTTCT-3'
YFP_Reverse	5'-GACGTTGTGGCTGTTGTAGTTGTA-3'

Supplementary Table 4. ASO sequences with detailed chemical modifications:

Name	Base/Backbone Modifications
Ube3a-ATS-targeting ASO	+A*+A*+C*+T*c*a*t*a*c*a*c*a*c*c*a*a*t*+T*+A
Ube3a-targeting ASO #1	/52MOErA/*/i2MOErA/*/i2MOErG/* /i2MOErC/*T*G* T*G*G* C*C*A* T*T*/i2MOErC/* /i2MOErG/*/i2MOErG/*/32MOErT/
Ube3a-targeting ASO #2	/52MOErC/*/i2MOErA/*/i2MOErT/* /i2MOErT/*C*T* C*C*G* A*A*T* C*T*/i2MOErG/* /i2MOErG/*/i2MOErT/*/32MOErC/
Ube3a-targeting ASO #3	/52MOErC/*/i2MOErG/*/i2MOErT/* /i2MOErA/*/i2MOErT/*A* G*C*C* A*C*C* G*T*C* /i2MOErA/*/i2MOErT/*/i2MOErA/* /i2MOErC/*/32MOErT/
Ube3a-targeting ASO #4	/52MOErC/*/i2MOErC/*/i2MOErT/* /i2MOErT/*/i2MOErC/*C* T*G*T* T*T*T* C*A*T* /i2MOErT/*/i2MOErT/*/i2MOErG/* /i2MOErT/*/32MOErA/
Ube3a-targeting ASO #5	/52MOErT/*/i2MOErT/*/i2MOErT/* /i2MOErG/*T*T* G*C*A* A*T*A* G*G*/i2MOErC/* /i2MOErT/*/i2MOErT/*/i2MOErG/* /32MOErA/
Ube3a-targeting ASO #6	/52MOErA/* /i2MOErT/* /i2MOErT/* /i2MOErMeC/* /i2MOErG/* G* C* T* A* G* C* T* T* C* A* /i2MOErA/* /i2MOErT/* /i2MOErG/* /i2MOErT/* /32MOErMeC/
Non-targeting control ASO	/52MOErG/*/i2MOErC/*/i2MOErG/* /i2MOErA/*/i2MOErC/*T* A*T*A* C*G*C* G*C*A* /i2MOErA/*/i2MOErT/*/i2MOErA/* /i2MOErT/*/32MOErG/
