# Supplementary material for: A dual-reporter mouse for therapeutic discovery in Angelman syndrome
Source: JCI Insight. 2026 Feb 3;11(5):e197028. doi: 10.1172/jci.insight.197028 (PMC13041673; doi:10.1172/jci.insight.197028)

Full unedited blots for Figure 2C

Sample order	Genotype
1	wild-type
2	matINSG
3	wild-type
4	matINSG
5	wild-type
6	matINSG
7	wild-type
8	matINSG
9	wild-type
10	matINSG

UBE3A Antibody Millipore Sigma, SAB1404508 – Chemiluminescence

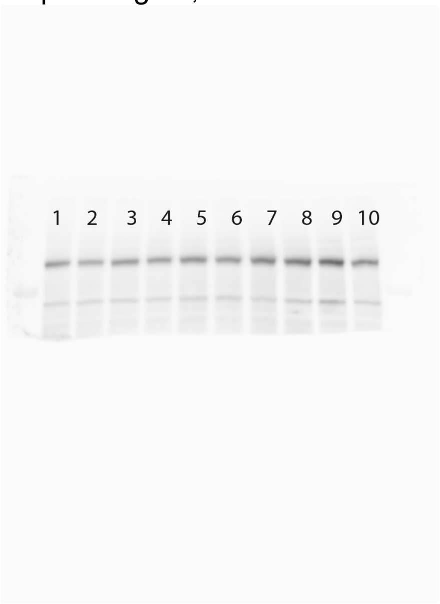

Visible light showing Precision Plus Protein™ All Blue Prestained Protein Standards

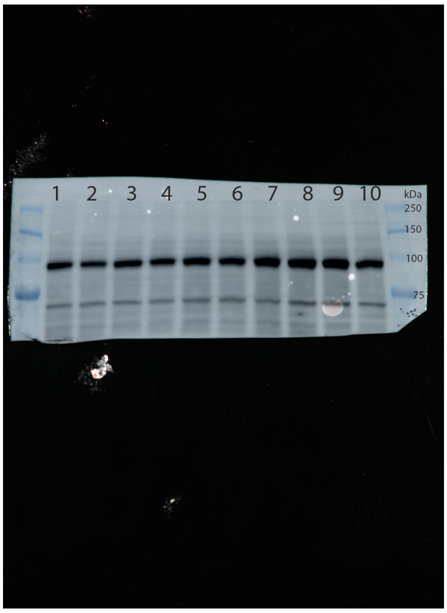

GAPDH Antibody Millipore Sigma, MAB374 – Chemiluminescence

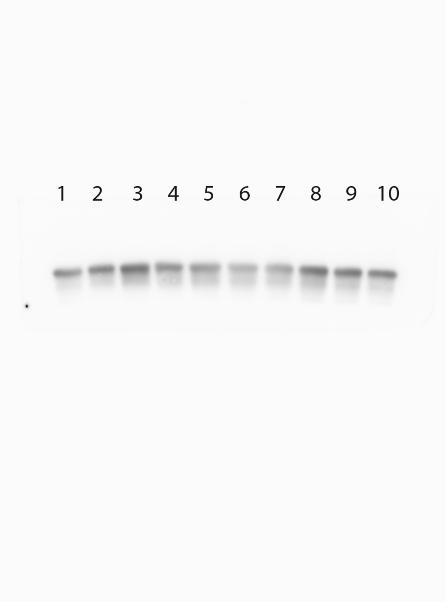

Visible light showing Precision Plus Protein™ All Blue Prestained Protein Standards

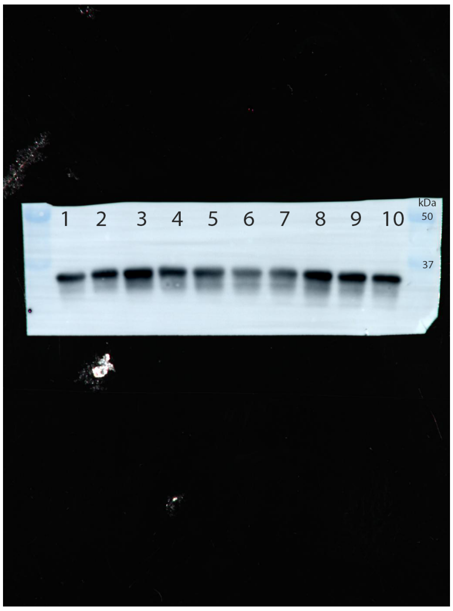

Sample order	Genotype
1	wild-type
2	patINSG
3	wild-type
4	patINSG
5	wild-type
6	patINSG
7	wild-type
8	patINSG
9	wild-type
10	patINSG

UBE3A antibody Millipore Sigma, SAB1404508 – Chemiluminescence

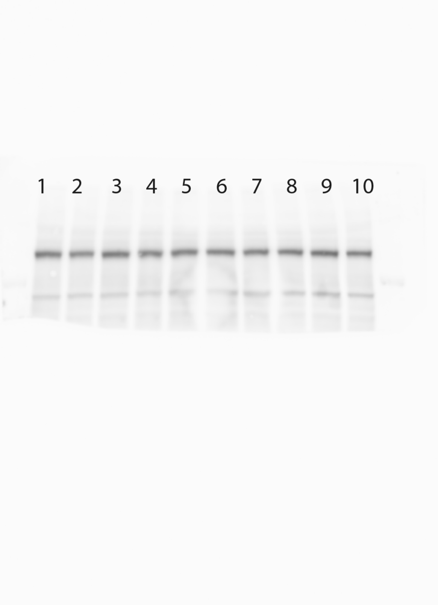

Visible light showing Precision Plus Protein™ All Blue Prestained Protein Standards

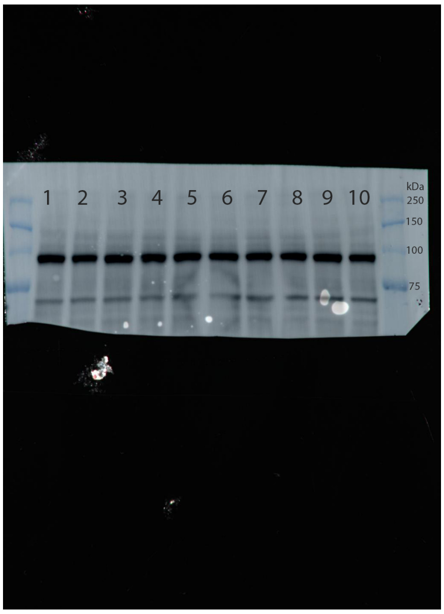

GAPDH Anitbody Millipore Sigma, MAB374 – Chemiluminescence

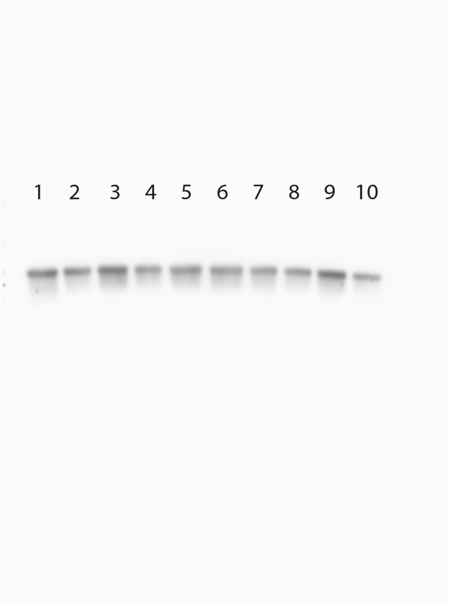

Visible light showing Precision Plus Protein™ All Blue Prestained Protein Standards

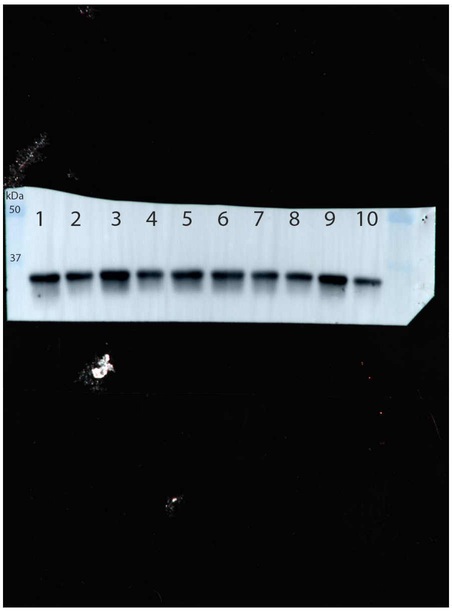

Full unedited blots for Figure 12F

Experiment I

Sample order	Sample name
1	NTC ASO
2	ASO #3
3	ASO #5
4	ASO #2
5	ASO #6
6	ASO #1
7	ASO #4
8	not included

UBE3A Antibody Millipore Sigma, SAB1404508 - Chemiluminescence

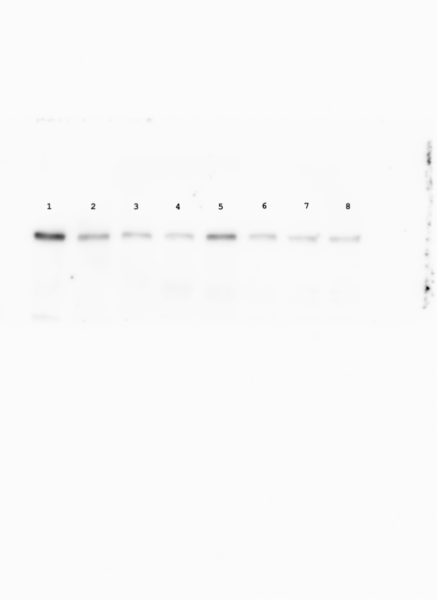

Visible light showing Precision Plus Protein™ All Blue Prestained Protein Standards

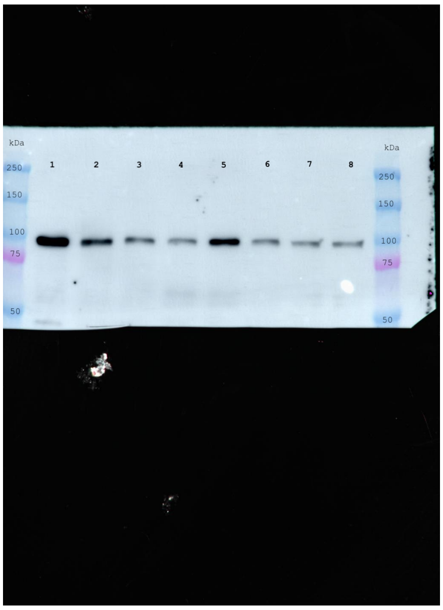

GAPDH Antibody Millipore Sigma, MAB374 – Chemiluminescence

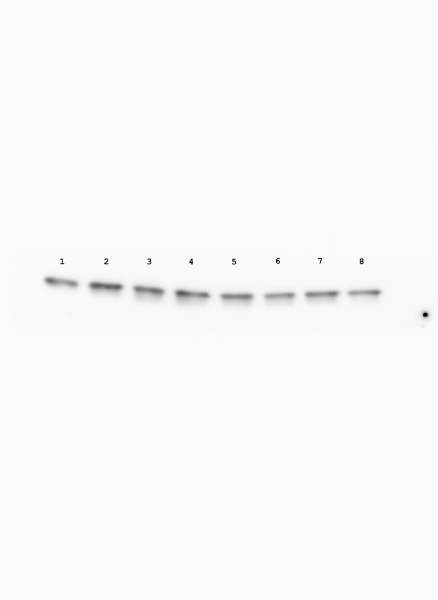

Visible light showing Precision Plus Protein™ All Blue Prestained Protein Standards

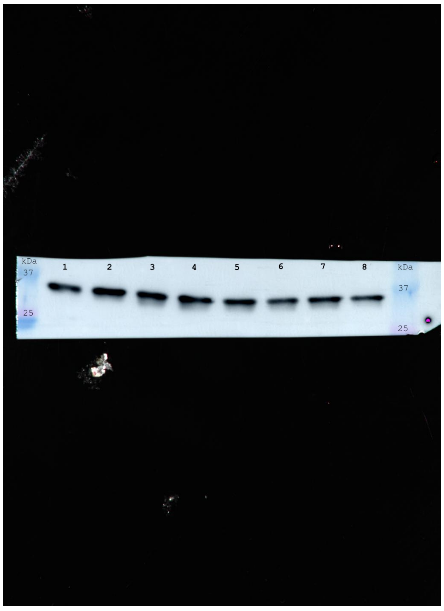

Experiment II

Sample order	Sample name
1	NTC ASO
2	ASO #3
3	ASO #5
4	ASO #2
5	ASO #6
6	ASO #1
7	ASO #4

UBE3A Antibody Millipore Sigma, SAB1404508 – Chemiluminescence

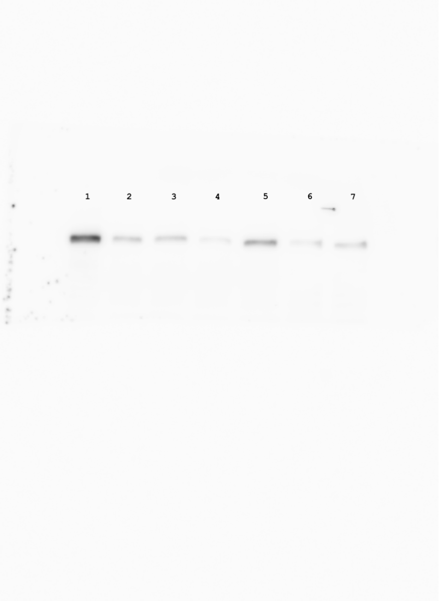

Visible light showing Precision Plus Protein™ All Blue Prestained Protein Standards

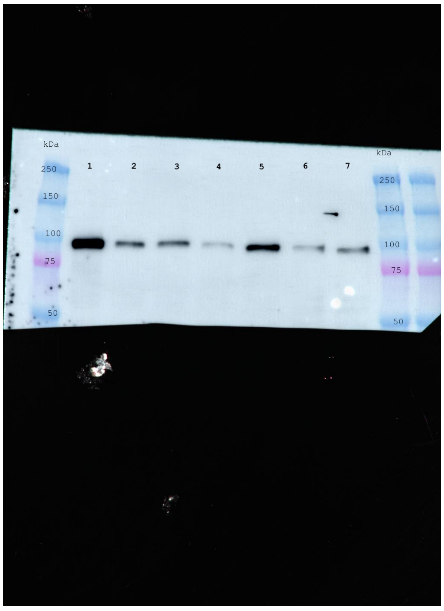

GAPDH Antibody Millipore Sigma, MAB374 – Chemiluminescence

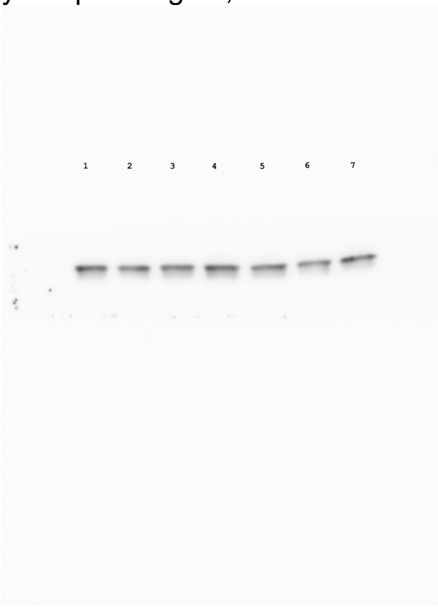

Visible light showing Precision Plus Protein™ All Blue Prestained Protein Standards

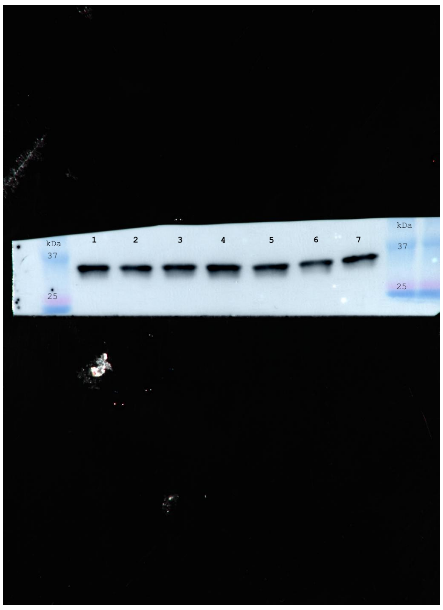

Experiment III

Sample order	Sample name
1	NTC ASO
2	ASO #3
3	ASO #5
4	ASO #2
5	ASO #6
6	ASO #1
7	ASO #4
8	not included

UBE3A Antibody Millipore Sigma, SAB1404508 – Chemiluminescence

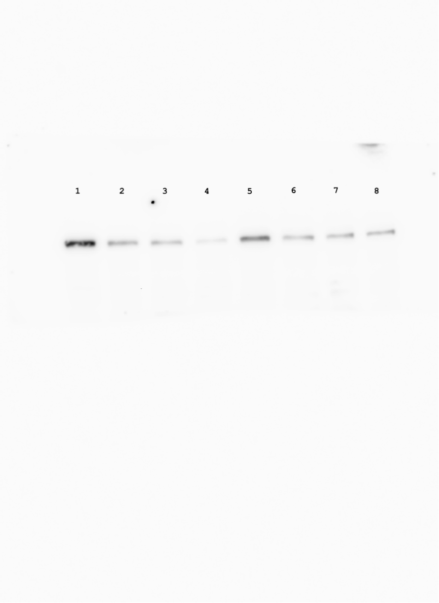

Visible light showing Precision Plus Protein™ All Blue Prestained Protein Standards

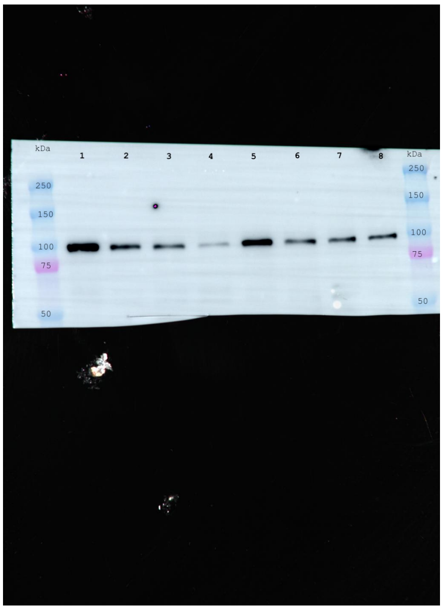

GAPDH Antibody Millipore Sigma, MAB374 – Chemiluminescence

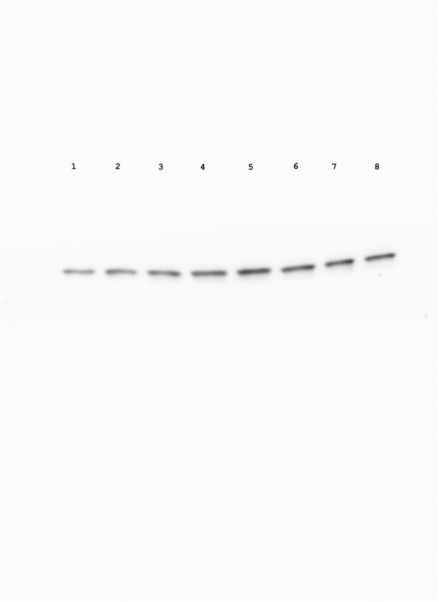

Visible light showing Precision Plus Protein™ All Blue Prestained Protein Standards

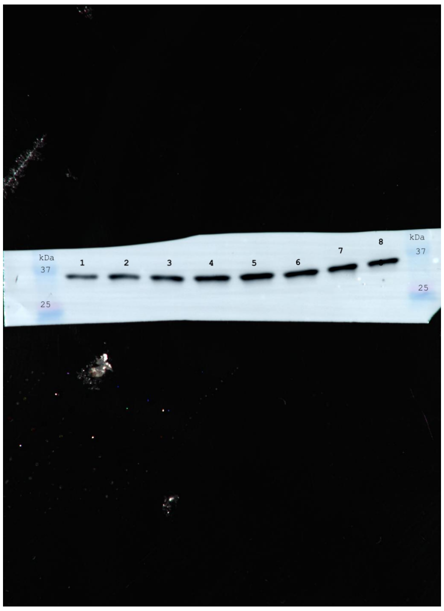

Supplement: Unedited blot and gel images [file jciinsight-11-197028-s309.pdf]
